# Three-Dimensional Impression of Biomaterials for Alveolar Graft: Scoping Review

**DOI:** 10.3390/jfb14020076

**Published:** 2023-01-29

**Authors:** Inês Francisco, Ângela Basílio, Madalena Prata Ribeiro, Catarina Nunes, Raquel Travassos, Filipa Marques, Flávia Pereira, Anabela Baptista Paula, Eunice Carrilho, Carlos Miguel Marto, Francisco Vale

**Affiliations:** 1 Institute of Orthodontics, Faculty of Medicine, University of Coimbra, 3000-075 Coimbra, Portugal; 2 Coimbra Institute for Clinical and Biomedical Research (ICBR), Area of Environment Genetics and Oncobiology (CIMAGO), Faculty of Medicine, University of Coimbra, 3000-075 Coimbra, Portugal; 3 Laboratory for Evidence-Based Sciences and Precision Dentistry, University of Coimbra, 3000-075 Coimbra, Portugal; 4 Centre for Innovative Biomedicine and Biotechnology (CIBB), University of Coimbra, 3000-075 Coimbra, Portugal; 5 Clinical Academic Center of Coimbra (CACC), 3030-370 Coimbra, Portugal; 6 Institute of Integrated Clinical Practice, Faculty of Medicine, University of Coimbra, 3004-531 Coimbra, Portugal; 7 Institute of Experimental Pathology, Faculty of Medicine, University of Coimbra, 3004-531 Coimbra, Portugal

**Keywords:** printing, three-dimensional, alveolar bone grafting, bone regeneration, bone substitutes

## Abstract

Craniofacial bone defects are one of the biggest clinical challenges in regenerative medicine, with secondary autologous bone grafting being the gold-standard technique. The development of new three-dimensional matrices intends to overcome the disadvantages of the gold-standard method. The aim of this paper is to put forth an in-depth review regarding the clinical efficiency of available 3D printed biomaterials for the correction of alveolar bone defects. A survey was carried out using the following databases: PubMed via Medline, Cochrane Library, Scopus, Web of Science, EMBASE, and gray literature. The inclusion criteria applied were the following: *in vitro*, *in vivo*, *ex vivo*, and clinical studies; and studies that assessed bone regeneration resorting to 3D printed biomaterials. The risk of bias of the *in vitro* and *in vivo* studies was performed using the guidelines for the reporting of pre-clinical studies on dental materials by Faggion Jr and the SYRCLE risk of bias tool, respectively. In total, 92 publications were included in the final sample. The most reported three-dimensional biomaterials were the PCL matrix, β-TCP matrix, and hydroxyapatite matrix. These biomaterials can be combined with different polymers and bioactive molecules such as rBMP-2. Most of the included studies had a high risk of bias. Despite the advances in the research on new three-dimensionally printed biomaterials in bone regeneration, the existing results are not sufficient to justify the application of these biomaterials in routine clinical practice.

## 1. Introduction

Craniofacial defects can originate from an array of etiological factors including congenital malformations, trauma, infection, rejection or implant failure, infection of bone graft, osteomyelitis, or surgical removal of tumors [1,2,3]. The craniofacial bone can also be impacted by systemic conditions such as osteodegenerative illnesses such as osteoporosis and arthritis, other impactful conditions include osteogenesis imperfecta and bone fibrous dysplasia [4]. All these conditions will compromise functional aspects such as phonation, mastication, and swallowing, which in turn affect the patient’s quality of life [5,6]. The two most common craniofacial bone defects are cancer of the head and neck and cleft lip and palate (CLP) [5,6,7,8,9]. CLP is a multifactorial pathology with several genetic and epigenetic factors as well as environmental factors such as geographical location, socioeconomical factors, and race [10,11]. In an attempt to minimize anomalies resulting from CLP, multidisciplinary treatment is initiated from birth and carries on into adulthood in order to achieve optimal results [12].

During the mixed dentition stage, individuals with CLP may require a secondary alveolar bone graft. During this period, this approach can result in relevant improvements such as closure of oronasal fistulae, stabilization of the two maxillary segments, and enhanced support of the alar base, which, in turn, will improve nasal and labial symmetry [13,14]. The secondary alveolar bone graft was introduced by Boyne and Sands in 1972 and it is currently regarded as the gold standard with the iliac crest being the most frequently chosen donor location [13]. In order to assert the proper timing to perform this procedure, the upper canine should have two thirds of its root developed which usually occurs between the ages of 9 and 11 [13].

The autologous bone graft can present with a variety of setbacks including limited amount of grafted bone, immune response risks, procedure time, and heavy costs. Additionally, a year after the procedure, bone reabsorption will happen in 40% of cases creating the need for re-intervention [15,16]. The main donor sites of autologous bone in craniomaxillofacial surgery are iliac crest graft and calvarial graft, but intraoral graft is also a possibility [17]. Currently, regenerative medicine has been established as a viable alternative in treatment of bone defects including CLP [18,19,20,21]. This approach can modulate the bone regeneration process and inflammation and enhance the healing process. Various biomaterials have been developed with the intent of overcoming the limitations of conventional bone grafts [22], such as heterologous or homologous bone graft [23,24]. These substituting materials can be used on their own or combined with an autologous bone graft and/or matrices. The most recognized tissue regeneration approach in the literature in the treatment of alveolar bone defects is bone morphogenetic protein 2 [25,26]. This approach provides comparable outcomes concerning bone volume, filling, and height to the gold standard technique with the iliac crest bone graft [26].

**Figure 1:**
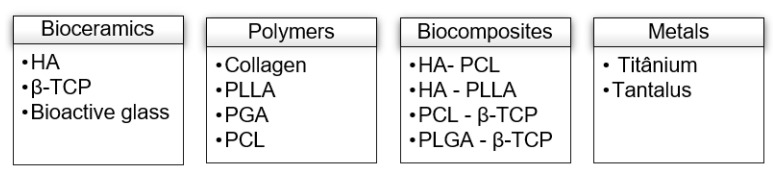
Most used matrices in bone regeneration. HA—Hydroxyapatite; β-TCP—β-tricalcicum-phosphate; PLLA—Polylactic acid; PGA—Glycolic acid; PCL—Polycaprolactone.

The matrices (Figure 1) are a subtract that allow for cell differentiation and proliferation. Their biocompatibility, biodegradability, osteoconduction, and mechanical properties are characteristics which can influence the success rate of the bone regeneration process [27].

These matrices can be three-dimensional (3D) printed enhancing its adaptation to the bone defect. With the use of 3D technologies, these matrices can be created and adapted according to the specific needs of each patient by changing their internal and external structures whilst using different materials [27,28].

The most commonly used matrices in bone defect treatment are bioceramic and are usually made out of hydroxyapatite (HA) or β-tricalcicum-phosphate (β-TCP). These materials are highly biocompatible and with osteoinductive abilities while also promoting rapid bone formation [29]. Despite a general increase of interest regarding 3D printed biomaterials in recent years, a comprehensive study regarding the general effectiveness of these biomaterials is lacking. To clarify this, we conducted a scoping review to assess the effectiveness of 3D printed biomaterials in the treatment of alveolar defects, which would be helpful for readership since it synthesizes what we know and the best future clinical approach in a single paper. Moreover, this knowledge will allow sustaining the realization of new future clinical studies. The aim of this paper is to put forth an in-depth review regarding the clinical efficiency of available 3D printed biomaterials for the correction of alveolar bone defects.

## 2. Materials and Methods

### 2.1. Study Research and Selection Strategy

Literature research was conducted on the PubMed data base via Medline, Cochrane Library, Web of Science Core Collection, EMBASE, and in gray literature. The last search was done, independently, on the 15th of August 2022 by two researchers.

A combination of Medical Subject Headings (Mesh) along with free text words were used in each of the databases (Appendix A). The following language filters were used: Portuguese, English, Spanish, and French. No filters were used regarding date of publication.

Two researchers initially scrutinized the articles independently by title and abstract. Subsequently, the articles were evaluated according to their full integral text; if doubts arose regarding the inclusion of a certain article, a third researcher was consulted.

The considered studies had to comply with the following inclusion criteria: *in vitro*, *in vivo*, *ex vivo*, and clinical studies; and studies that assessed bone regeneration resorting to 3D printed biomaterials. The exclusion criteria applied were as follows: non-clinical studies and every other type of research (editorials, academic books, and reports); case reports or descriptive studies; duplicated studies; studies with incomplete data; and studies that merely reported on the characterization of a new biomaterial without reporting on bone regeneration rates.

### 2.2. Data Extraction

After the eligibility process, the articles were sorted into different categories according to the type of study: *in vitro*, *in vivo*, *ex vivo*, or clinical. From each selected article, the following information was extracted: authors, date of publication, study design, experimental and control group, evaluation time, bone regeneration assessment method, results, and main conclusions.

### 2.3. Risk of Bias

The bias risk of the *in vitro* studies was obtained using the Faggion Jr. norms for pre-clinical studies regarding dental materials [30]. For the *in vivo* studies, the bias risk tool from the Systematic Review Centre for Laboratory Animal Experimentation (SYRCLE) was used.

## 3. Results

### 3.1. Study Selection

**Figure 2:**
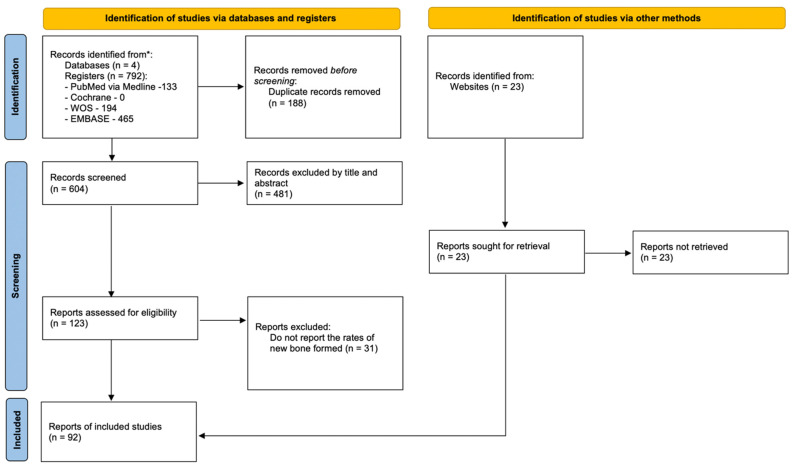
Flowchart.

The initial search, performed on the previously mentioned databases, gathered 792 studies. After removing duplicates, 604 studies were scrutinized according to title and abstract. Afterwards, all references deemed irrelevant for this systematic review were excluded, resulting in 123 potentially relevant studies. Given that 31 articles did not report bone regeneration rates, only 92 references were included in the final sample. The identification, screening and eligibility process is summarized in the flow chart (Figure 2).

### 3.2. Characteristics of the Included Studies

#### 3.2.1. *In Vitro* Studies

Fifty-one articles analyzed the properties of biomaterials *in vitro*. The year of publication ranged from 2015 to 2022, with the exception of one study conducted in 2006 [31]. The most commonly used biomaterial in the control group was PCL matrix, followed by β-TCP and PLLA. Osteogenic activity through alkaline phosphatase was the most widely used method to assess bone regeneration, having been described in 26 articles. Seventy- two studies evaluated bone regeneration through the expression of osteogenesis-related genes. Only one study [32] reported the release rate of growth factors. On the other hand, one study [33] evaluated the porosity of the matrix and found that the presence of nanotubes is associated with more favorable results for osteogenesis when compared to larger pores. Table 1 summarizes the results of the *in vitro* studies included in this systematic review.

#### 3.2.2. *In Vivo* Studies

*In vivo* bone regeneration was evaluated in 75 articles, published between 2015 and 2022, in various animal species, such as New Zealand rabbits, beagle dogs, and rat models. The number of animals used in each study ranged from 3 to 120, with seven articles not reporting the sample size [30,31,82,83,84,85].

The most commonly used biomaterial in the control group was β-TCP matrix, followed by PCL matrix. Regarding the evaluation method, microcomputerized tomography was the most used followed by histology. Other methods used were real-time polymerase chain reaction [42,86] and immunohistochemistry [87].

The most refracted matrices were PCL, β-TCP, and HA. In seven articles, the matrix of the experimental group contained bone morphogenetic protein-2 (BMP-2) [28,42,45,49,88,89]. Bone regeneration was superior in all experimental groups, with the exception of three articles [90,91,92], which found similar values between the control and experimental group. Regarding secondary outcomes, Van Hede et al. [73] analyzed matrix geometry, and found that the gyroid geometry results in better outcomes when compared to the orthogonal one. Chang et al. [43] found that combining HA matrix with an oxidized RGD peptide in a high stiffness matrix may be advantageous for maxillofacial regeneration when compared to low stiffness matrices.

Table 2 summarizes the results of the *in vivo* studies included in the present systematic review.

### 3.3. Synthesis of Quantitative Evidence

In the various studies evaluated, many different biomaterials are described. The most referenced biomaterial was β-tricalcium phosphate (β -TCP), used in 16 *in vitro* studies and 27 *in vivo* studies. The second most referenced biomaterial was polycaprolactone (PCL), mentioned in 16 *in vitro* studies and 20 *in vivo* studies. Hydroxyapatite (HA) was the third most used biomaterial, in 7 *in vitro* and 16 *in vivo* studies. There are other biomaterials/biomolecules that were used in more than 3 studies, namely: decellularized extracellular matrix (dECM), human recombinant bone protein type 2 (RhBMP-2), collagen, polylactic acid (PLLA), polylactic acid-co-glycolic acid (PLGA), calcium sulfate (SC), and different types of hydrogel (e.g., bone-derived extracellular matrix, β-TCP, cell-laden, nanocomposite, MicroRNA). All other biomaterials are mentioned only in a few studies, generating a multitude of results, which makes them difficult to analyze, and, consequently, to draw conclusions (Table 3).

The most used evaluation method was different in *in vitro* and *in vivo* studies. In the first ones, the most frequent methods were the following: determination of osteogenesis-related gene expression by qRT-PCR (27 studies), and the evaluation of alkaline phosphatase activity, a mineralization precursor protein, by p-nitrophenol assay (9 studies), and by a staining assay with the AKT assay kit (7 studies). In *in vivo* studies, radiological methods such as micro-CT (57 studies) and histological methods (56 studies) are the most used (Table 4).

The most used 3D printing technique mentioned in both types of studies is extrusion-based 3D printing (23 *in vitro* studies and 27 *in vivo* studies). However, there are other techniques used simultaneously in *in vitro* and *in vivo* studies, namely: fused deposition modeling (6 and 10, respectively), stereolithography (2 and 7, respectively), and laser sintering technique (3 in both). Other techniques are used, but only occasionally in 1 or 2 studies (Table 5).

### 3.4. Risk of Bias

The risk of bias of the *in vitro* and *in vivo* studies is summarized in Table 6 and Table 7, respectively. Regarding *in vitro* studies, none described the methodology to implementation sample. All *in vivo* studies also lacked information regarding sample allocation, allocation randomization process methodology, implementation, and protocol. All but three of the articles disclose information regarding study financing.

Regarding *in vivo* studies, most of the studies have serious methodological flaws, leaving out pivotal information such as sequence generation, allocation concealment, and blinding. Only six studies specify investigator blindness as a factor during outcome assessment. Lastly, seven other studies report no additional bias sources.

## 4. Discussion

The aim of the present systematic review was to report the current state of the art regarding the clinical efficiency of available 3D printed biomaterials for the correction of alveolar bone defects. Although the quantitative analysis of the results could not be executed due to the heterogeneity of the studies, the qualitative analysis allowed for a better understanding and evaluation of the published studies.

The conventional technique requires an autologous graft of cancellous bone and is considered the gold standard [13]. However, with the limited offer of donor bone as well as the bone reabsorption rate due to its adaptability to the defect site, a re-intervention may be necessary [15,16]. In an attempt to diminish these limitations, studies have been carried out in order to explore different approaches that can accelerate bone formation, reduce bone reabsorption and improve soft tissue scarring. 3D printed biomaterials can be specifically made to adapt to the bone defect site; this has led to an increase in studies regarding this topic over the last five years [27,28].

Out of the 75 *in vivo* studies included, 17 evaluated the efficiency of the PCL matrix [32,35,37,50,51,52,53,54,55,62,63,65,66,73,77,81,120]. This biomaterial is the most well reviewed biomaterial in literature due to its high biocompatibility, durability and subsequent extensive use [37]. Despite its low degradation rate, the PCL matrix is limited in terms of cellular adhesion and osteogenic differentiation, several authors [32,35,49,50,53,62] have suggested combining it with different polymers [37] and bioactive molecules such as rBMP-2, that promote proliferation and differentiation of mesenchymal stem cells into osteoblasts resulting in bone formation. Nonetheless, a recently published umbrella review regarding the efficiency of current approaches in regeneration of bone defects in non-syndromic patients with cleft palate concluded that rBMP2 seems to provide results similar to the iliac crest bone graft in terms of bone volume and vertical dimension [121]. Another limitation of the PCL matrix is its low hydrophilia [52], which can be amended when the matrix is combined with a hydrophilic material such as β -TCP [35,66,77] or polydopamine [37]. With the addition of graphene, the PCL matrix increases its capacity to induce the secretion of growth factors that boost angiogenesis [56].

The β-TCP matrix was reportedly used in 12 *in vitro* and 30 *in vivo* studies. This calcium phosphate bioceramic presents ideal biocompatibility and osteoconductivity [36,64,85]. In addition to those characteristics, the β-TCP matrix also contains components similar to the bone tissue apatite along with a good balance between reabsorption and degradation during bone formation. Despite all these attributes, the osteogenic abilities of this biomaterial showed subpar results when used in large bone defects [35,48,64] and thus falling short when compared to the autologous bone graft [70]. 

The hydroxyapatite matrix is one of the most referenced bioceramics in *in vivo* studies. When combined with β-TCP this matrix becomes highly biocompatible and with a great osteointegration rate [88,90,123]. However, more studies are required in order to fully understand the macro-design that can optimize bone regeneration [90]. Since the bone formation process involves the immune system, this can be modulated by biomaterials such as esphingosine-1-phosphate (S1O) which has been linked to the β-TCP matrix. This sphingolipid has been shown to increase the expression of genes related to osteogenesis, such as osteoporin (OPN), transcribing factor 2 related to a runt (RUNX2), and osteocalcin (OCN) [36]. In addition to this, the combination of β-TCP with strontium oxide (SrO), sillica (SiO_2_), magnesium (MgO), and zinc (ZnO) also proved to be effective in bone regeneration due to alterations in the physical and mechanical properties of the matrix [48]. 

Regarding PRF, this biomaterial can improve the reconstruction of the alveolar cleft. It is prepared from centrifuged autologous blood formed by a fibrin matrix that contains platelets, white blood cells, growth factors and cytokines. These factors may promote the uniqueness and differentiation pathways of osteoblasts, endothelial cells, chondrocytes, and various sources of fibroblasts, stimulating the regenerative capacity of the periosteum. Furthermore, the fibrous structure of PRF acts as a three-dimensional fibrin scaffold for cell migration [16]. In this way, PRF can be used with a bone substitute, allowing wound sealing, homeostasis, bone union, and graft stability [16]. In contrast, BMP-2 is usually applied in alloplastic bone grafts or scaffolding and is an effective inducer of bone and cartilaginous formation. Its application avoids the limitation of autologous bone grafts, which may be related to the shorter operative and hospitalization time. However, it has some adverse effects, such as nasal stenosis and localized edema at the graft site [26].

Another promising candidate for bone regeneration is the pure Zn L-PBF porous scaffold [74]. It presented relatively adjusted deterioration rates and mechanical strength for bone implants. Furthermore, they also showed well *in vitro* cytocompatibility with MC3T3-E1 cells and osteogenic capacity for hBMMSCs. The *in vivo* implantation results showed that pure Zn scaffolds have potential for applications in large bone defects with osteogenic properties [74].

Additionally, the microstructure of the matrices such as porosity, pore size, and structure play a very important role in cell viability and bone growth [115]. In contrast to traditional methods, the development of three-dimensional printing allows for the control of the microstructure. Therefore, a wide variety of materials and techniques are available to optimize the matrix [124]. Shim et al. reported that porosity affects osteogenesis, with matrices with 30% porosity showing better osteogenic capacity than groups with 50% and 70% porosity [115]. Regarding pore size, the literature suggests that the ideal size should be between 400 to 600 μm [63,103,111]. Finally, the pore configuration should also be considered in terms of the dynamic stability of the matrix. Recently, matrices with hierarchical structures have been studied. Zhang et al. demonstrated that tantalum matrices with hierarchical structures exhibited excellent hydrophilicity, biocompatibility, and osteogenic properties [33]. However, in the future, additional *in vivo* studies are required as to understand what structure the matrix should present in order to find a balance between cell viability and mechanical properties of the biomaterial, optimizing bone regeneration.

This systematic review presents some limitations that may alter the interpretation of the results, namely: (1) some of the included studies present a small sample size with only three animals; (2) the included studies present high risk of bias; (3) lack of evaluation of variables that interfere with bone regeneration, such as the position of the teeth in the bone graft, the width of the defect, the volume of grafted bone and the experience of the clinician; (4) absence of clinical studies; (5) heterogeneity of the studies in terms of matrix typology and follow-up used may difficult outcome assessment. Due to the heterogeneity in the methodology of the included studies, most of the studies selected in this systematic review were classified as having a high risk of bias, which may decrease the certainty of the results. According to the risk of bias analysis, the analyzed parameters with the highest risk of bias were sample allocation, allocation randomization process methodology, implementation, and protocol. These factors must be considered when figuring out the results of this review. The methodology of the several studies evaluated is very different and is not described enough, which makes their effective comparison impossible. Since there are numerous types of biomaterials/biomolecules and various combinations between them, future studies should define the most appropriate methodology, creating guidelines for its implementation and subsequent comparison. 

In addition, future studies should be calibrated in order to use similar parameters and protocols, providing stronger evidence, focusing on the most described materials, namely β-tricalcium phosphate, polycaprolactone, hydroxyapatite with decellularized extracellular matrix (dECM), human recombinant bone protein type 2 (RhBMP-2), collagen, polylactic acid (PLLA), poly(lactic acid-co-glycolic acid (PLGA), and calcium sulfate (CS). Moreover, these promising materials should be evaluated and compared to each other in a single study in order to obtain more effective and clinically applicable conclusions. In the future, additional studies should be performed, more specifically blinded randomized studies with increased control of possible bias sources namely, the randomization process, concealment of the investigators of the experimental groups and description of the limitations of the studies. Moreover, the cost-effectiveness of the proposed new regenerative strategies should be evaluated, as it plays a crucial role in clinical decision making in healthcare systems, especially public institutions.

Lastly, future systematic reviews focused on 3D biomaterials should include only the most referenced evaluation and printing techniques. Therefore, for *in vitro* systematic reviews, the authors should compare PCL, b-TCP, RhBMP-2, and HA biomaterials created by extrusion printing, fused deposition, stereolithography, or laser sintering techniques. The chosen evaluation methodology should be gene expression by qRT-PCR and alkaline phosphatase activity. On the other hand, for *in vivo* systematic reviews, the authors should analyze the same biomaterials and the same technique printing, but the evaluation methodology should be based on radiology imaging and histology.

## 5. Conclusions

The most reported three-dimensional biomaterials were the PCL matrix, β-TCP matrix, and hydroxyapatite matrix. Despite the advances in the research on new three-dimensionally printed biomaterials in bone regeneration, the existing results are not sufficient to justify the application of these biomaterials in routine clinical practice.

## Figures and Tables

**Table 1:** Characteristics of *in vitro* studies.

Authors, Year	Control Group	Experimental Group	Cell Culture	Evaluation Time	Bone Regeneration Evaluation Method	Printing Technique	Results	Conclusion
Alksne M. et al., 2020 [34]	PLLA scaffold	-PLLA scaffold + HA 10% -PLLA scaffold + BG	Rat dental pulp stem cells DPSCs	1, 7, 10 days	ALP activity evaluated by p-nitrophenol assay and osteogenesis-related gene expression quantified with qPCR	Extrusion-based bioprinting	The scaffold with BG shows better osteoinductive properties than that with HA	PLLA+BG scaffold is promising in bone regeneration
Bae E. et al., 2018 [35]	PCL/ β TCP scaffold	-dECM/PCL/ β TCP scaffold -dECM/PCL/β TCP/ rhBMP-2 scaffold	MC3T3-E1 cells (mouse preosteoblasts)	1, 3, 5, 7, 14, 21 e 28 days	ALP activity evaluated by p-nitro phenol assay	Extrusion-based bioprinting	The dECM/PCL/β TCP/rhBMP-2 scafffold showed higher FA expression than the other scaffolds	dECM can be combined with rhBMP-2 to enhance bone regeneration
Cao Y. et al., 2019 [36]	β TCP scaffold	S1P coated β -TCP scaffold	RAW264.7 cells (macrophage cells) + BMSC cells (Rat bone marrow stromal cells)	3 days	Osteogenic-related gene expression quantified by qRT-PCR	3D-Bioplotter	S1P-coated β-TCP scaffold increased the expression of osteogenesis-related genes	S1P-coated β-TCP scaffold promotes bone regeneration
Chen Y. et al., 2018 [37]	Cells cultured on the tissue culture plate without scaffold	-PDASC/PCL scaffold -PDASC/PCL/hydrogel scaffold	RFP-HUVEC cells + Wharton’s jelly mesenchymal stem cells (WJMSCs)	1, 3, 7 days	Osteogenic-related protein secretion determined by an ELISA	Inkjet-based bioprinting	PDASC/PCL/hydrogel scaffold showed higher expression of osteogenesis-related proteins	PDASC/PCL/hydrogel scaffold can be applied in bone regeneration
Chiu Y. et al., 2019 [38]	SC scaffold	SrSC scaffold	Mouse fibroblasts L929 cell line	1, 3, 7 days	Expression levels of osteogenic-related proteins via western blot	3D printing	Increased mineralization in the SrSC scaffold	SrSC scaffold is promising in bone regeneration
Cooke M. et al., 2020 [39]	DPSCs without dexamethasone and β-glycerol-2-phosphate in a LayFomm scaffold	DPSCs with dexamethasone and β-glycerol-2-phosphate in a LayFomm scaffold	Dental Pulp Stem Cells (DPSCs)	21 days	Histological evaluation of the calcified matrix formed	Fused deposition modeling	DPSCs with dexamethasone and β-glycerol-2-phosphate in a LayFomm scaffold are able to form mineralized matrix	LayFomm is a promising scaffold for craniofacial bone regeneration
Dai Q. et al., 2021 [40]	0Cu-BG	-2Cu-BG -5Cu-BG -10Cu-BG	Mouse bone mesenchymal stem cells (BMSCs)	1, 3, 7 days	Osteogenesis-related gene expression quantified by qRT-PCR	Extrusion-based hydrogel 3D printing	-In the presence of Cu there is increased differentiation of stem cells -The highest osteogenesis-related gene expression occurred in the group with 2Cu	Bioactive glass containing Cu promotes stem cell proliferation and regenerated bone tissue quality
Dubey N. et al., 2020 [41]	Hydrogel scaffold	Hydrogel scaffold with MgP	Dental pulp stem cells (DPSCs)	7, 14 days	Osteogenesis-related gene expression quantified by qRT-PCR	Microvalve Bioprinting	The scaffold with MP increased the expression of osteogenesis-related genes	The presence of MP in the scaffold can increase bone formation
Fahimipour F. et al., 2019 [42]	βTCP/collagen/heparin scaffold	βTCP/collagen/heparin/ BMP-2 scaffold	Mesenchymal stem cells (MSCs)	7, 14 days	Osteogenesis-related gene expression quantified by qRT-PCR	Extrusion-based bioprinting	The presence of BMP-2 led to an increased expression of osteogenesis-related genes	The β TCP/collagen/heparin/ BMP-2 scaffold is effective and should be explored for other bioactive molecules
Gómez-Cerezo M. et al., 2020 [43]	BG/ PVA scaffold	-BG/PVA-2d -BG/PVA-30d	rBMSCs (femora marrow rats)	3, 7 days	Osteogenesis-related gene expression quantified by qRT-PCR	Extrusion-based additive manufacturing method	The BG/PVA-2d scaffold showed higher expression of genes related to osteogenesis	Immersion of the BG/PVA scaffold in PBS improves the osteogenic properties of the scaffolf
Han L. et al., 2021 [44]	PLGA scaffold without Fe coating	Fe-coated PLGA scaffold	rBMSCs	1, 2, 3, 7 e 14 days	Osteogenesis-related gene expression quantified by qRT-PCR	3D printing	Fe-coated PLGA scaffold increased expression of osteogenesis-related genes	3D scaffolds with nanocomposites enhance osteogenic differentiation of mesenchymal stem cells
Huang K. et al., 2021 [45]	SC/ CS scaffold	SC/CS/BMP-2 scaffold	Human dental pulp stem cells (hDPSCs)	3 days	ALP activity via western blot	Extrusion-based bioprinting	The SC/CS/BMP-2 scaffold showed higher levels of osteogenic ALP activity	SC/CS/BMP-2 scaffold is promising for bone regeneration
Jeong J. et al., 2020 [46]	100% gelatin scaffold	Gelatin and β-TCP scaffold	MC3T3-E1 preosteoblast cells	7 days	ALP activity evaluated by p-nitro phenol assay	Extrusion-based bioprinting	Scaffolds with 60% β-TCP and 40% gelatin show the best cellular activity	Scaffolds with 60% β-TCP and 40% gelatin are a bone substitute with potential
Kao C. et al., 2015 [47]	PLLA scaffold	PLLA/PDA scaffold	Human adipose-derived stem cells (hADSCs)	3, 7 days	ALP activity evaluated by p-nitro phenol assay	Stereolithography	ALP activity was higher in the PLLA/PDA scaffold	PDA is a promising tool in bone regeneration
Ke, D. et al., 2018 [48]	β TCP scaffold	β-TCP, SrO, SiO_2_, MgO and ZnO scaffold	Human preosteoblast cell line (hFOB 1.19)	3, 9 days	Osteogenesis-related gene expression quantified by qRT-PCR	Fused deposition modeling	The β TCP/MgO and β TCP/SiO_2_ scaffolds demonstrated the highest expression of osteogenesis-related genes	The β TCP/MgO and β TCP/SiO_2_ scaffolds are promising for bone regeneration
Kim B. et al., 2018 [49]	PCL scaffold	PCL + BMP-2 + HA scaffold	Human bone marrow-derived mesenchymal stem cells (hMSCs)	7 days	ALP activity	3D printing	The PCL+ BMP-2 + HA scaffold increased the activity of FA	Osteogenic properties are superior in the PCL + BMP-2 + HA scaffold
Kim J. et al., 2017 [50]	MgP ceramic scaffold	MgP/KR-34893 scaffold	Human bone marrow-derived mesenchymal stem cells (hMSCs)	1, 3, 5, 7 days	Osteogenesis-related gene expression quantified by qRT-PCR	Extrusion-based bioprinting	MgP/ KR-34893 scaffold increased the expression of osteogenesis-related genes	Addition of KR-34893 promotes greater osteogenic differentiation
Lee S. et al., 2018 [51]	PCL scaffold	PCL/BFP-1 scaffold	Human tonsil-derived mesenchymal stem cells (hTMSCs)	7, 14 days	ALP activity evaluated by p-nitro phenol assay	Fused deposition modeling	The PCL/BFP-1 scaffold was shown to have the highest osteogenic efficacy	The PCL/BFP-1 scaffold is promising is efficient in bone regeneration
Li J. et al., 2017 [52]	PCL scaffold	-PCL and traditional PRP scaffold -PCL/PRP scaffold freeze-dried	Human dental pulps DPSCs	7, 14 days	Osteogenesis-related gene expression quantified by qRT-PCR	Fused deposition modeling	The freeze-dried PCL/PRP scaffold increased the expression of osteogenesis-related genes	The freeze-dried PCL/PRP scaffold promotes greater bone formation
Li Y. et al., 2019 [53]	PCL scaffold	PCL/Asp@Lipo/BFP-1 scaffold	Human mesenchymal stem cells (hMSCs)	7, 14, 21 days	ALP activity quantified by AKP assay kit	3D printing, method not described	The 3:7 Asp@Lipo/BFP-1 ratio was shown to have the highest osteogenic efficacy	This is a promising scaffold for craniofacial bone regeneration
Lin Y. et al., 2019 [54]	Culture of hSF-MSCs	PEEK scaffold with hSF-MSCs	Human mesenchymal stem cells (MSCs)	1, 4, 7, 14, 21 days	Osteogenesis-related gene expression quantified by qRT-PCR	Laser sintering technique	hSF-MSCs proliferate in the PEEK scaffold	PEEK/ hSF-MSCs is a promising scaffold in bone regeneration
Lin YH. et al., 2017 [55]	PCL scaffold	-PCL/10%SC scaffold -PCL/30%SC scaffold -PCL/50%SC scaffold	Wharton’s Jelly mesenchymal stem cells (WJMSCs)	7 days	Osteogenesis-related gene expression quantified by qRT-PCR	Extrusion-based bioprinting	PCL/50% scaffold induced higher expression of osteogenesis-related genes	PCL/SC scaffold shows favorable osteoconductive properties and is a promising biomaterial for bone regeneration
Lin YH. et al., 2019 [56]	Neat graphene	GCP scaffold	Human Wharton’s Jelly mesenchymal stem cells (WJMSCs)	3, 7 days	Osteogenesis-related gene expression via western blot	Extrusion-based bioprinting	GCP scaffold induced higher expression of osteogenesis-related proteins	GCP scaffold promotes osteogenesis
Martin V. et al., 2019 [57]	PLLA/col scaffold	-PLLA/col/MH scaffold -PLLA/col/MH/HA scaffold	Human bone marrow-derived mesenchymal stem cells (hMSCs)	5, 10, 15 days	Osteogenesis-related gene expression quantified by qRT-PCR	Extrusion-based bioprinting	-Incorporation of HA increased the expression of osteogenesis-related genes -The combination of HA and MH resulted in increased osteogenic activity	PLLA/col/MH/HA scaffolds stimulates osteogenesis and has a therapeutic action against Staphylococcus aureus, which makes it promising in bone regeneration
Mi X. et al., 2022 [58]	HA/Sodium alginate scaffold	HA/Sodium alginate/Ti_3_C_2_ MXene	Bone mesenchymal stem cells (BMSCs)	7, 14 days	Osteogenesis-related gene expression quantified by qRT-PCR	Extrusion-based bioprinting	The experimental scaffold exhibited excellent biocompatibility, promoted cell proliferation and upregulated osteogenic gene expression	Ti_3_C_2_ MXene composite 3D-printed scaffolds are promising for clinical bone defect treatment
Miao Y. et al., 2019 [59]	Hidrogel scaffold	Hidrogel scaffold with FP	Mesenchymal stem cells hMSCs	7, 14 days	Osteogenesis-related gene expression quantified by qRT-PCR	Nanosheets via liquid phase stripping method	The addition of FP increased the osteogenesis-related gene expression	Hydrogel and FP scaffold may constitute a good strategy for bone regeneration
Midha S. et al., 2018 [60]	Bioactive glass 45S5	-Bioactive Silk Fibrin Glass with Strontium -Strontium-free fibrin silk bioactive glass	TVA-BMSC cell line	21 days	Osteogenesis-related gene expression quantified by qRT-PCR	Extrusion-based bioprinting	The strontium group showed higher expression of osteogenesis-related genes	Silk fibrin bioactive glass promising for bone formation
Pan T. et al., 2022 [61]	Hydrogel scaffold combined with miRNA	Hydrogel scaffold with miRNA and 0.25;1;2.5% GTA	Mesenchymal stem cells hMSCs	7, 14, 21, 28, 42 days	Osteogenesis-related gene expression quantified by qRT-PCR	Extrusion-based bioprinting	The scaffold with 1% GTA presented the best characteristics for bone regeneration	The hydrogel/miRNA/1%GTA scaffold is promising for bone regeneration
Park J. et al., 2015 [32]	PCL scaffold	PCL/VEGF/BMP-2 scaffold	Human dental pulp stem cells (DPSCs)	7, 14 days	Growth Factor Release Rate	Extrusion-based bioprinting	Bone regeneration was superior in the scaffold with growth factors	Scaffolds with growth factors are a promising alternative
Park S. et al., 2020 [62]	PCL scaffold	PCL/ β TCP scaffold	Mouse preosteoblast cell line MC3T3-E1	7 days	ALP activity quantified by AKP assay kit	Selective laser sintering	The PCL/ β TCP scaffold showed higher ALP	The addition of β TCP to the PCL scaffold is advantageous for bone regeneration
Ratheesh G. et al., 2021 [63]	FDM-manufactured PCL scaffold	PCL scaffold by FDM and MEW	Human joint tissue explant cells	3, 7, 21 days	Osteogenesis-related gene expression quantified by qRT-PCR	MEW and FDM	The PCL scaffold by FDM/MEW showed higher expression of genes related to osteogenesis	MEW membrane promotes a more favorable environment for osteogenic differentiation
Remy M. et al., 2021 [64]	β TCP/miRNA scaffold	βTCP/miRNA/collagen scaffold	Primary human BMSCs (hBMSCs)	7 days	Osteogenesis-related gene expression quantified by qRT-PCR	Stereolithography	The β TCP/miRNA/collagen scaffold showed higher expression of osteogenesis-related genes	The β TCP/miRNA/collagen scaffold is promising in the treatment of bone defects
Roh H. et al., 2016 [65]	PCL/HA scaffold	PCL/HA and MgO scaffold	Pre-osteoblast (MC3T3-E1) cells	1, 3 e 5 days	Osteogenesis-related gene expression quantified by qRT-PCR	Extrusion-based bioprinting	The addition of MgO increased the osteogenesis-related gene expression	PCL/HA/MgO scaffold is promising for bone formation
Shim J. et al., 2017 [66]	Collagen membrane	-PCL scaffold -PCL/ β-TCP scaffold	NIH3T3 (mouse fibroblasts) + MC3T3-E1 (mouse preosteoblasts	1, 4, 7, 14 days	Proliferation rates of fibroblasts	Multilayer membrane 3D printing	Osteogenic differentiation was higher in the PCL/ β-TCP scaffold	The PCL/ β-TCP scaffold shows good results in bone regeneration
Shuai C. et al., 2020 [67]	HA/PLLA scaffold	HA/PLLA e PGA scaffold	MG-63 human osteoblast-like cells	8 weeks	Formation of mineralized matrix	Laser-assisted bioprinting	The HA/PLLA/PGA scaffold has proven to be a suitable environment for cell culture	The HA/PLLA/PGA scaffold is capable of bone and vascular formation
Tcacencu I. et al., 2018 [68]	-SW ceramic glass-ceramic scaffold -PLLA scaffold	AW/PLLA scaffold	Bone marrow-derived stromal cells (BMSCs)	7, 14 days	ALP activity evaluated by p-nitro phenol assay	Indirect 3D printing/fused filament fabrication	The AW scaffold showed higher activity of ALP	AW scaffold has good osteoconductive properties
Tsai C. et al., 2019 [69]	Ti scaffold	Ti scaffold with Mg- SC and CH	Human Wharton’s Jelly mesenchymal stem cells (WJMSCs)	3, 7 days	ALP activity quantified by AKP assay kit	Selective laser melting	The Ti/Mg-CS/CH scaffold increased the activity of ALP	Ti/Mg-CS/CH scaffold increases osteogenesis
Umeyama R. et al., 2020 [70]	β TCP scaffold	β TCP/RCP scaffold	Bone marrow cells isolated from C57BL/6J mice	4, 7, 14 days	Osteogenesis-related gene expression quantified by qRT-PCR	3D printing	The β TCP/RCP showed higher Osteogenesis-related gene expression	The addition of RCP is efficient in bone regeneration
Wang P. et al., 2021 [71]	PLLA scaffold	-Sodium hydroxide conditioned PLLA scaffold -PlA scaffold with PDA conditioned with NaOH	Bone marrow stromal cells (BMSCs)	7, 14 days	ALP activity evaluated by p-nitro phenol assay	Fused deposition modelling	The PLLA scaffold with PDA conditioned with sodium hydroxide showed higher activity of ALP	PLLA scaffold with PDA conditioned with sodium hydroxide is promising for bone formation
Wang S. et al., 2020 [72]	PCL e Bio-Oss scaffold	PCL/ Bio-Os/NaOH scaffold	Human bone marrow-derived mesenchymal stem cells (hBMMSCs)	7, 14 days	ALP activity quantified by AKP assay kit	Fused deposition modeling	The PCL/ Bio-Oss/NaOH scaffold increased ALP	PCL/ Bio-Oss/NaOH scaffold is promising for bone formation
Weinand C. et al., 2006 [31]	β TCP scaffold	β TCP/type I collagen in hydrogel scaffold	Bone-marrow-derived differentiated mesenchymal stem cells (MSCs)	6 weeks	Osteogenesis-related gene expression quantified by qRT-PCR	Inkjet-based bioprinting	Osteogenesis-related gene expression was higher in β TCP/type I collagen scaffold	The β TCP/type I collagen scaffold is promising for bone formation
Wu Y. et al., 2019 [73]	SC and PCL scaffold	dECM/SC/PCL scaffold	Human Wharton’s Jelly mesenchymal stem cells (WJMSCs)	6 h, 1 and 7 days	Osteogenesis-related gene expression quantified by qRT-PCR	Extrusion-based bioprinting	The dECM/SC/PCL scaffold increased the expression of osteogenesis-related genes	dECM/SC/PCL scaffold is promising for bone regeneration
Xia D. et al., [74]	Zinc scaffold	Pure zinc porous scaffold	Mouse pre-osteogenic cells (MC3T3-E1 cell line)	7, 14 days	Osteogenesis-related gene expression quantified by qRT-PCR	Laser powder bed fusion technology	Pure zinc porous scaffold showed higher expression of osteogenesis-related genes	Pure Zn porous scaffolds with customized structures represent a promising biodegradable solution for treating large bone defect
Xu Z. et al., 2019 [75]	β TCP/PLGA scaffold	β TCP/PLGA/PDA scaffold	Mouse pre-osteogenic cells (MC3T3-E1 cell line)	7, 14 days	ALP activity quantified by AKP assay kit	Extrusion-based bioprinting	β TCP/PLGA/PDA scaffold increased ALP activity	The addition of PDA promotes osteogenesis
Xu Z. et al., 2022 [76]	β TCP/PVA scaffold	β TCP/ PVA/ dipyridamole scaffold	Mouse pre-osteogenic cells (MC3T3-E1 cell line)	7, 14 days	ALP activity quantified by ALP assay kit	Extrusion-based bioprinting	The β TCP/ PVA/ dipyridamole scaffold increased ALP	β TCP/PVA/dipyridamole composite scaffolds have brilliant potential in new bone formation as a suitable alternative
Yun S. et al., 2021 [77]	PCL scaffold	dECM/β TCP/PCL scaffold	MG63 cells	1, 3, 5, 7, 14 days	ALP activity quantified by AKP assay kit	Extrusion-based bioprinting	The dECM/ β TCP/PCL scaffold increased ALP	The dECM/β TCP/PCL scaffold was shown to have superior osteogenic potential
Zamani Y. et al., 2021 [78]	β TCP/PLGA scaffold by solvent/leach technique	3D printed β TCP/ PLGA scaffold	MC3T3-E1 pre-osteoblasts	14 days	ALP activity evaluated by p-nitro phenol assay	Extrusion-based bioprinting	The β TCP/ PLGA 3D scaffold showed higher ALP activity	The β TCP/ PLGA 3D scaffold is more favorable for bone formation
Zhang Y. et al., 2019 [79]	β TCP/PLGA scaffold	β TCP/PLGA/OG/BMP-2 scaffold	rMSCs	1, 4, 7 days	ALP activity evaluated by p-nitro phenol assay	Extrusion-based bioprinting	β TCP/ PLGA/ OG/ BMP-2 scaffold increased ALP activity	β TCP/PLGA/OG/BMP-2 is a promising scaffold for bone regeneration
Zhang Z. et al., 2021 [33]	p-Ta scaffold	p-Ta-nt scaffold	MC3T3-E1 preosteoblasts	7 days	Osteogenesis-related gene expression quantified by qRT-PCR	3D printing laser melting system	Tantalum scaffold with nanotubes showed higher expression of osteogenesis-related genes	Tantalum scaffold with nanotubes holds promise for bone formation
Zhao N. et al., 2017 [80]	β TCP scaffold e HÁ scaffold	HA/β TCP scaffold with different HA compositions (0.20, 0.40, 0.60, 0.80 and 1.00)	Bone mesenchymal stem cells (BMSCs)	1, 4, 7 days	Osteogenesis-related gene expression quantified by qRT-PCR	3D printing	40% HA scaffold showed higher osteogenic capacity	HA / β TCP scaffold is promising for bone formation
Zhong L. et al., 2020 [81]	PCL scafold	-PCL/DCPD scaffold -PCL/DCPD and nanoZIF-8 scaffold	Bone mesenchymal stem cells (BMSCs)	25 days	Osteogenesis-related gene expression quantified by qRT-PCR	Extrusion-based bioprinting	PCL/DCPD/nanoZIF-8 scaffold increased osteogenesis-related gene expression	The PCL/DCPD/ nanoZIF-8 scaffold is a bone substitute with potential

3D—three dimensional, Asp@Lipo—aspirin loaded liposomes, AW—apatite-volastonite, BFP-1—bone forming peptide 1, BG—bioactive glass, BG/PVA-2d—bioactive glass/polyvinyl acid in phosphate-salt buffer 2 days, BG/PVA-30d—bioactive glass/polyvinyl acid in phosphate buffered saline 30 days, Bio-Oss—deproteinized bovine bone mineral, BMP-2—bone morphogenetic protein type-2, CH—chitosan, CS—calcium sulfate, Cu—copper, Cu (10Cu-BG) —bioactive glass with 15% copper, Cu (2Cu-BG) —bioactive glass with 7% copper, Cu (5Cu-BG)—bioactive glass with 10% copper, DCPD—calcium phosphate dihydrate, dECM—decellularized extracellular matrix, FA—alkaline phosphatase, Fe—iron, FDM—fusion and deposition method, FP—black phosphorus, GCP—calcium silicate with graphene/polycaprolactone, GTA—glutaraldehyde, HA—hydroxyapatite, hSF-MSCs—synovial mesenchymal stem cells, KR-34893—bioactive organic compound, MEW—melt electrospinning writing, MgO—magnesium oxide, MgP—magnesium phosphate, MH—minocycline, miRNA—microRNA, nanoZIF-8—nanoscale zeolitic imidazolate framework-8, NaOH—sodium hydroxide, nt—nanotubes, OG—graphene oxide, PCL—polycaprolactone, PDA—polydopamine, PDASC—polydopamine modified calcium silicate, PEEK—polyetheretherketone, PGA—polyglycolic acid, PLGA—poly(lactic acid-co-glycolic acid), PLLA—polylactic acid, PLLA/col—polylactic acid/collagen, PRP—platelet-rich plasma, p-Ta—porous tantalum, PVA—polyvinyl acid, PBS—phosphate-saline buffer, RCP—recombinant collagen peptide, rhBMP-2—human recombinant bone protein type 2, S1P—sphingosine-1-phosphate, SC—calcium silicate, SiO_2_—silica, SrO—strontium oxide, SrSC—calcium strontium silicate, Ti—titanium, VEGF—endothelial growth factor, ZnO—zinc oxide, β TCP—β-tricalcium phosphate.

**Table 2:** Characteristics of *in vivo* studies.

Authors, Year	Sample Size (n)/Animal Model	Control Group	Experimental Group	Evaluation Time	Bone Regeneration Evaluation Method	Printing Technique	Results	Conclusion
Bae E. et al., 2018 [35]	n = 28 male SD rats	Group without scaffold (n = 7)	-Group with scaffold PCL/β-TCP (n = 7) -Group with scaffold dECM/ PCL/β-TCP (n = 7) -Group with scaffold dECM/ PCL/β-TCP/rhBMP-2 (n = 7)	4 weeks	μ-CT, histology	Extrusion-based 3D printing	Bone formation was significantly higher in the group with the dECM/ PCL/β-TCP/rhBMP-2 scaffold (43.32% ± 7.63)	The dECM/PCL/β-TCP/rhBMP-2 scaffold promotes bone regeneration
Bekisz J. et al., 2018 [93]	n = 10 defects in 5 Finn Dorset sheeps	Group with HA/ β-TCP/collagen scaffold (n = 5)	Group with HA/ β-TCP/collagen/dipyridamole 100 μM scaffold (n = 5)	3, 6 weeks	μ-CT, histology	Extrusion-based 3D printing	Osteogenesis was higher in the experimental group at 3 and 6 weeks	Dipyridamole significantly increases the capacity for bone regeneration
Bose S. et al., 2018 [85]	Male SD rats	Group with β-TCP scaffold	Group with β-TCP/curcumin/PCL/PEG scaffold	6 weeks	Histology	Binder jetting	The formation of mineralized bone, after 6 weeks, was higher in the experimental group (44.9%)	The β-TCP/curcumin/PCL/PEG scaffold is an excellent candidate for bone regeneration
Chang P. et al., 2021 [94]	n = male SD rats	Group without scaffold (n = 6)	-Group with HA scaffold (n = 6) -Group with HA and nonoxidized RGD peptide with lower stiffness (n = 6) -Group with HA scaffold and nonoxidized RGD peptide with osteoid-like stiffness (n = 6) -Group with HA scaffold and oxidized RGD peptide with osteoid-like stiffness (n = 6)	7, 28 days	μ-CT, Histology	Extrusion-based 3D printing	-Limited bone regeneration was observed in the group with HA scaffold and nonoxidized RGD peptide with osteoid-like stiffness -There was greater bone formation at both time points in the group with HA scaffold and oxidized RGD peptide with osteoid-like stiffness	The combination of HA with oxidized RGD peptide in a osteoid-like stiffness scaffold may be beneficial for maxillofacial regeneration
Chen M. et al., 2021 [95]	n = 32 male SD rats	Group without scaffold (n = 8)	-Group with PRF (n = 8) -Group with PCL scaffold (n = 8) -Group with PRF/PCL scaffold (n = 8)	4, 8 weeks	μ-CT, histology	Fused deposition modeling	-More mineralization was observed in the groups with scaffold at 4 and 8 weeks -The presence of PRF did not influence bone formation	The use of PCL scaffolds enhances bone formation
Chiu Y. et al., 2019 [38]	New Zealand rabbits	Group with SC scaffold	Group with SrSC scaffold	4, 8 weeks	μ-CT, histology	3D printing	There is more bone and vascular formation in the experimental group at 4 (26.3 ± 1.9%) and 8 weeks (45.7 ± 6.2%)	SrSC scaffold enhances bone regeneration
Cooke M. et al., 2020 [39]	n = 12 male SD rats	Group without LayFomm scaffold (n = 6)	Group with LayFomm scaffold (n = 6)	6 weeks	μ-CT	Fused deposition modeling	-The mechanical properties of the scaffold are a limitation in large defects -There is greater production of mineralized tissue in the group with LayFomm scaffold	LayFomm scaffold is promising in craniofacial regeneration
Dai Q. et al., 2021 [40]	n = 40 defects in 20 male SD rats	Defects without scaffold	-Defects with Gel/SF scaffold -Defect with Gel/SF/0Cu-BG scaffold -Defect with Gel/SF/2Cu-BG, Gel/SF/5Cu-BG and Gel/SF/10Cu-BG scaffold	4, 8 weeks	μ-CT, histology	Extrusion-based hydrogel 3D printing	-The group with the Gel/SF/2Cu-BG scaffold produced the largest number of blood vessels -At 4 weeks, the Gel/SF/5Cu-BG scaffold presented the highest bone formation -At 8 weeks, the Gel/SF/2Cu-BG scaffold presented the highest bone formation	The most effective scaffold for bone regeneration was Gel/SF/5Cu-BG
Diomede F. et al., 2018 [96]	n = 24 male Wistar rats	Group with PLLA scaffold (n = 4)	-Group with PLLA scaffold and hGMSCs (n = 4) -Group with PLLA/EV scaffold (n = 4) -Group with PLLA/hGMSCs/EVs scaffold (n = 4) -Group with PLLA/PEI-EVs scaffold (n = 4) -Group with PLLA/EIP-EVs/hGMSCs scaffold (n = 4)	6 weeks	μ-CT	Fused deposition modeling	The groups with the PLLA/PEI-EVs and PLLA/PEI-EVs/ hGMSCs scaffolds demonstrated greater bone regeneration and better osteogenic properties with 12.27% and 9.71% new bone formation, respectively	PLLA scaffolds conjugated with PEI-EVs are promising in bone regeneration
Dubey N. et al., 2020 [41]	n = 16 male Fisher 344 rats	Group without scaffold (n = 4)	-Group with PTFE (n = 4) -Group with ECM scaffold (n = 4) -Group with ECM/MgP scaffold (n = 4)	4, 8 weeks	μ-CT, histology	Microvalve 3D printing	-The control group and the PTFE membrane group showed little bone formation -In the group with the ECM/AMP scaffold, a greater bone density was observed at 4 and 8 weeks than in the other groups	The presence of MgP enhances bone regeneration and is promising for bone defect repair
El-Habashy S. et al., 2021 [97]	n = 24 New Zealand rabbits	Grupo without scaffold (n = 6)	-Group with polyvinyl acid scaffold (n = 6) -Group with HA scaffold (n = 6) -Group with HA/PCL scaffold (n = 6)	2, 6 weeks	μ-CT	Extrusion-based 3D printing	The HA/PCL scaffold showed better biocompatibility, osteoconduction and osteogenic properties at both time points	HA/PCL scaffold is promising in bone defect repair
Fahimipour F. et al., 2019 [42]	n = 15 male Fisher 344 rats	-Group with β-TCP/collagen/heparin scaffold (n = 5) -Group with β-TCP/collagen/BMP-2 scaffold (n = 5)	Group with β TCP/collagen/heparin/BMP-2 scaffold (n = 5)	6 weeks	Histology, qPCR	Inkjet-based 3D printing	The experimental group showed superior osteogenic differentiation and increased bone formation	The bioactive molecule BMP-2 increases scaffold efficiency in bone regeneration
Fama C. et al., 2020 [98]	n = 14 defects in 7 rats	------	-Group with porous β-TCP scaffold (n = 7) -Group with non-porous β-TCP scaffold (n = 7)	8 weeks	μ-CT, histology	3D printed scaffolds	-In the groups with the non-porous scaffold, greater bone formation was observed -The porous scaffold exhibited greater soft tissue volume	Non-porous scaffold enhances bone regeneration
Guéhennec L. et al., 2019 [90]	n = 12 male SD rats	Group with HA scaffold (n = 6)	Group with HA:60- β TCP:40 scaffold (n = 6)	3, 6 months	μ-CT, histology	Stereolithography	The groups showed similar amount of bone formed 3 and 6 months after intervention	Calcium phosphate scaffolds have good osseointegration and biocompatibility and should be studied to achieve the ideal level of bone regeneration
Han L. et al., 2021 [44]	n = 14 male SD rats	Group without scaffold (n = 6)	-Group with Fe-coated PLGA scaffold (n = 4) -Group with PLGA scaffold without Fe coating (n = 4)	8 weeks	μ-CT	3D printing	The amount of bone formed was higher in the Fe-coated scafold, followed by the uncoated scaffold	Magnetic scaffold promotes bone regeneration
He M. et al., 2021 [99]	n = 12 female SD rats	Group without scaffold (n = 4)	Group with hydrogel scaffold with PPG-1.5 (n = 4)	4 weeks	Histology	Extrusion-based 3D printing	In the group with the PPG-1.5 scaffold, bone formation was higher	PPG-1.5 scaffold provides good mechanical support for bone growth
Huang K. et al., 2021 [45]	n = 6 male New Zealand rabbits	Group with SC/CS scaffold (n = 3)	Group with SC/CS/BMP-2 scaffold (n = 3)	4 weeks	μ-CT, histology	Extrusion-based 3D printing	The MS/CS/BMP-2 scaffold promoted greater vascular and bone growth	The MS/CS scaffold can act as a carrier for BMP-2 and is an ideal biomaterial for bone regeneration
Ishack S. et al., 2017 [88]	n = 15 murine rats	Group with HA/β-TCP scaffold (n = 5)	-Group with HA/ β-TCP/dipyridamole scaffold (n = 5) -Group with HA/ β-TCP/BMP-2 scaffold (n = 5)	2, 4, and 8 weeks	μ-CT, histology	Extrusion-based 3D printing	The experimental groups demonstrated greater bone formation at 2, 4 and 8 (47.5 ± 5% for dipyridamole and 48.3 ± 4% for BMP-2) weeks compared to the control group	Addition of dipyridamole and BMP-2 to HA/ β-TCP scaffold promotes bone formation
Jeong J. et al., 2020 [47]	n = 20 male SD rats	Group with 100% gelatin scaffold (n = 4)	Group with gelatin scaffold (40%) and β-TCP (60%)	4 weeks	μ-CT	Extrusion-based 3D printing	The scaffold with β-TCP induced significantly more bone formation	The presence of β-TCP provides a more favorable environment for bone formation
Jia L. et al., 2021 [100]	n = 18 male SD rats	Group without scaffold (n = 6)	-Group with PLLA scaffold (n = 6) -Group with PLLA scaffold and iron oxide (n = 6)	4 weeks	μ-CT	Direct ink writing technique	Iron oxide scaffold promoted bone formation and altered the composition of the oral microbiom	Iron oxide scaffold can be used to treat bone defects of the palate
Johnson Z. et al., 2021 [101]	n = 6 yorkshire farm pigs	Group without scaffold (n = 3)	Group with HA/ β-TCP scaffold (n = 3)	8 weeks	μ-CT, histology	Stereolithography	Bone regeneration was superior in the group with the HA/ β-TCP scaffold	HA/ β-TCP scaffold seems to be effective in bone regeneration
Ke D. et al., 2018 [48]	n=12 rat distal femoral defects	Group with β TCP scaffold	Group with β TCP, SiO_2_, and MgO scaffold	8, 12, 16 weeks	Histology	Fused deposition modeling	-At week 8, both groups had similar amounts of mineralized bone -The experimental group presented greater bone formation at 12 and 16 weeks	The β TCP/Si/Mg scaffold significantly increased osteogenesis compared to the control group matrix, making it promising for bone regeneration
Kim J. et al., 2020 [102]	n = 12 adult male beagles	Group without scaffold (n = 4)	-Group with β-TCP/ HA scaffold without synthetic polymer (n = 4) -Group with β-TCP/ HA scaffold with synthetic polymer (n = 4)	4, 8 weeks	Histology, imagiologia	Stereolithography	The group with the β-TCP/ HA scaffold without the synthetic polymer showed greater bone regeneration in both moments	The β-TCP/ HA scaffold without the synthetic polymer can be used for bone regeneration
Kim J. et al., 2017 [50]	n = 24 male SD rats	Group without scaffold (n = 6)	-Group with MgP scaffold (n = 6) -Group with MgP scaffold and 5 μM of KR-34893 (n = 6) -Group with MgP scaffold and 25 μM KR-34893 (n = 6)	4, 8 weeks	μ-CT, histology	Extrusion-based 3D printing	-The number of osteoclasts decreases in the presence of KR-34893 - Bone formation is higher in groups with scaffold containing KR-34893	The compound KR-34893 is gradually released from the scaffold, increasing bone volume
Lee D. et al., 2018 [103]	n = 12 male SD rats	------	Group with HCCS-PDA scaffold and 250 μm pore size (n = 6) Group with HCCS-PDA scaffold and 500 μm pores (n = 6)	8 weeks	μ-CT, histology	Digital light processing-type 3D printing system	-Limited bone growth was observed in the group with the 250 μm pore scaffold -The group with the 500 μm pore scaffold showed greater bone regeneration	The pore size of the HCCS-PDA scaffold that induces the most effective bone regeneration is 500 μm
Lee J. et al., 2021 [86]	n = 10 beagles	Group with PCL/ β-TCP/dECM scaffold (n = 5)	Group with PCL/ β-TCP/bdECM scaffold + ADSC injection (n = 5)	8 weeks	μ-CT, histology, qPCR	Fused deposition modeling	The experimental group demonstrated greater expression of genes related to osteogenesis and osteoblasts	Injection of stem cells derived from adipose tissue enhances ossification
Lee S. et al., 2019 [51]	n = 12 Male New Zealand white rabbits	Group with PCL scaffold (n = 3)	-Group with PCLD scaffold (n = 3) -Group with PCLDB100 scaffold (n = 3) -Group with PCLDB1000 scaffold (n = 3)	8 weeks	Histology, imagiologia	Fused deposition modeling	In the group treated with PCLDB1000 scaffold, a higher rate of bone formation and number of blood vessels was observed	PCLDB1000 scaffold is promising for bone regeneration
Lee SH. et al., 2019 [87]	New Zealand rabbits	Group with PCL scaffold	Group with PCL kagome-structure scaffold	4, 16 weeks	μ-CT, histology, immunohistochemistry	Extrusion-based 3D printing	The experimental group demonstrated bone formation at 4 and 16 weeks	The scaffold with kagome-structure can be applied in bone defect reconstruction
Liang T. et al., 2021 [104]	n = 9 beagles	Group without scaffold	-Group with HA/SA scaffold -Group with HA/SA/NG scaffold -Group with HA/SA/CGRP scaffold	1, 2, and 3 months	μ-CT	Micro extrusion 3D printing	-Greater bone growth was observed in the experimental groups at months 1, 2, and 3 -The groups with HA/SA/NG and HA/SA/CGRP scaffolds demonstrated greater osteogenic potential	-HA/SA scaffold is promising for bone regeneration -NG and CGRP may lead to increased bone proliferation
Li J. et al., 2017 [52]	n = 24 ratos machos SD	Grupo com matriz PCL (n = 8)	-Group with PCL matrix and traditional PRP (n = 8) -PCL matrix/PRP freeze-dried (n= 8)	2, 4, 8, 12 weeks	μ-CT, histology	Fused deposition modeling	-Addition of freeze-dried PRP to the PCL matrix promotes greater bone regeneration	Addition of freeze-dried PRP to the PCL matrix promotes greater bone regeneration
Li Y. et al., 2019 [53]	Male New Zealand rabbits	Group without scaffold	-Group with PCL/Asp@Lipo/BFP-1 scaffold -Group with PCL/Asp@Lipo scaffold -Group with PCL/BFP-1 scaffold	8 weeks	Histology	3D printing, method not described	The group treated with PCL/Asp@Lipo/BFP-1 scaffold showed greater bone formation, followed by the group treated with PCL/BFP-1	The hybrid scaffold PCL/Asp@Lipo/BFP-1 showed good osteogenic properties
Lim H. et al., 2020 [105]	n = 12 male New Zealand rabbits	-----	Group with HA/TCP scaffols with pores 0.8; 1.0; 1.2; 1.4 mm	4, 8 weeks	μ-CT	Digital light processing	-At week 4, larger pores result in greater bone formation -At week 8, there was no correlation between % bone formation and pore size	Pore size only influences bone regeneration in the initial phase
Lin YH. et al., 2019 [54]	n = 10 female New Zealand rabbits	Group without PEEK scaffold	-Group with PEEK scaffold and hSF-MSCs in standard culture medium -Group with PEEK scaffold + hSF-MSCs in osteogenic culture medium -Group with PEEK scaffold	4, 12 weeks	μ-CT, histology	Laser sintering technique	The largest volume of bone formed was observed in the group with PEEK scaffold + hSF-MSCs) in a standard culture medium at 4 and 12 weeks	The combination of PEEK scaffold + hSF-MSCs is effective in regenerating bone defects
Lin YH. et al., 2017 [56]	n = 12 New Zealand rabbits	Group with SC/PCL scaffold (n = 6)	Group with graphene/SC/PCL scaffold in a 10/40/50 ratio (n = 6)	4, 8 weeks	μ-CT, histology	Extrusion-based 3D printing	In the experimental group, the volume of bone formed was significantly higher at 4 and 8 weeks	PCL scaffolds containing graphene and calcium silicate are promising in bone regeneration
Liu A. et al., 2016 [106]	n = 20 male New Zealand rabbits	Group with β TCP scaffold (n = 10)	Group with akermanite scaffold (n = 10)	6, 12 weeks	μ-CT, histology	Extrusion-based 3D printing	-The percentage of bone formed at 6 and 12 weeks was significantly higher in the experimental group -The βTCP scaffold exhibited low mechanical properties	Akermanite scaffold is promising in bone regeneration
Lopez C. et al., 2019 [107]	n = 15 New Zealand rabbits	Group with β TCP scaffold (n = 5)	-Group with β-TCP and collagen scaffold (n = 5) -Group with β-TCP and collagen and dipyridamole scaffold (n = 5)	8 weeks	μ-CT, histology	Extrusion-based 3D printing	In the groups without dipyridamole, less bone growth and more residual scaffold was observed than in the group with dipyridamole	Dipyridamole significantly increased the bone regenerative capacity of the bioceramic scaffold
Mi X. et al., 2022 [58]	n = 36 male SD rats	Group without scaffold (n = 12)	-Group with HA/sodium alginate scaffold (n = 12) -Group with HA/sodium alginate/Ti_3_C_2_ MXene scaffold (n = 12)	4, 8 weeks	μ-CT, histology	Extrusion-based 3D printing	The group with the scaffold with Ti_3_C_2_ MXene promoted bone healing to a significantly greater degree than the other groups	The Ti_3_C_2_ MXene composite 3D-printed scaffolds are promising for clinical bone defect treatment
Miao Y. et al., 2019 [59]	Male Wistar rats	-Group without scaffold -Hydrogel scaffold group	Group with hydrogel scaffold and FP nanoparticles	3, 6, and 9 weeks	μ-CT, histology	Nanosheets via liquid phase stripping method	-The incorporation of FP promoted mineralization and reinforced the mechanical properties of the scaffold -Bone regeneration in the experimental group was superior at 3, 6, and 9 weeks	The hydrogel/FP scaffold can be applied in bone regeneration
Naudot M. et al., 2020 [108]	n = 22 male SD rats	Group with PCL scaffold (n = 11)	Group with PCL/HA/ BM-MSCs scaffold (n = 11)	2 months	μ-CT, histology	Electrospinning and electrospraying	The experimental group showed significantly higher bone formation over the two months	The combination of PCL scaffold with HA and BM-MSCs is promising for bone defect regeneration
Pan T. et al., 2022 [61]	n = 20 BALB/c rats	-Group without scaffold (n = 4) -Group with hydrogel scaffold combined with miRNA (n = 4)	-Group with hydrogel scaffold with miRNA and 0.25 GTA (n = 4) -Group with hydrogel scaffold with miRNA and 1 GTA (n = 4) -Group with hydrogel scaffold with miRNA and 2.5 GTA (n = 4)	2, 4, 8 weeks	μ-CT, histology	Extrusion-based 3D printing	Bone regeneration was significantly higher in the groups with 1GTA and 2.5GTA at 2, 4 and 8 weeks	The presence of miRNA and GTA induces osteogenesis, making this scaffold promising for the area of bone regeneration
Park S. et al., 2020 [62]	n = 8 defects in 4 male beagles	Defects in a PCL scaffold (n = 2)	-Defects with PCL/T50 scaffold (n = 2) -Defects with PCL/T0/B2 scaffold (n = 2) -Defects with PCL/T50/B2 scaffold (n = 2)	3 months	μ-CT	Selective laser sintering	-The volume of bone formed in defects with the PCL/T50 scaffold was significantly higher than with the PCL scaffols -In the scaffolds with rhBMP-2, bone regeneration was significantly higher	PCL/T50 scaffold is beneficial for transporting rhBMP-2 and regenerating bone in mandibular defects
Park J. et al., 2015 [32]	n = 30 BALB/c-nu/nu	Group with PCL scaffold (n = 10)	-Group with PCL/BMP-2 scaffold (n = 10) -Group with PCL/BMP-2/VEGF scaffold (n = 10)	4 weeks	Quantification of osteogenic genes in dental pulp stem cells	Extrusion-based 3D printing	Bone regeneration was faster in the vascularized scaffold	Vascularized scaffold is promising in bone regeneration
Pae H. et al., 2018 [109]	n = 10 male New Zealand rabbits	Group without scaffold	-Group with PCL scaffold -Group with PCL/10% β-TCP scaffold -Group with PCL/10% β-TCP and collagen membrane	2, 8 weeks	μ-CT	3D printing	Bone formation was only observed in the scaffolds containing β-TCP	Addition of β-TCP to the PCL scaffold increases osteoconductivity
Qiao S. et al., 2020 [110]	n = 30 female New Zealand rabbits	Group with Ti scaffold (n = 15)	Group with Ti scaffold modified by hydrogel with medium concentrations of silver nanoparticles (n = 15)	6, 12 weeks	μ-CT, histology	3D printing	The experimental group showed significantly higher bone regeneration at 6 and 12 weeks	Hydrogel-modified Ti scaffold with medium concentrations of silver nanoparticles is promising for treating bone defects
Qin H. et al., 2022 [111]	n = 24 male New Zealand white rabbits	----	-Group with magnesium-substituted calcium scaffold with 480 μm pore size -Group with magnesium-substituted calcium scaffold with 600 μm pore size -Group with magnesium-substituted calcium scaffold with 720 μm pore size	2,4,8, 12 weeks	μ-CT, histology	Digital light processing	There was a higher new bone ingrowth rate in the 600 μm group than the other two groups at 4–12 weeks post-implantation	The magnesium-substituted calcium scaffold with 600 μm pore size is promising to guide new bone ingrowth
Qin Y. et al., 2022 [112]	n = 10 male New Zealand rabbits	Group with pure Zn scaffolds (n = 10)	Group with Zn-1Mg porous scaffolds (n = 10)	6, 12 weeks	histology	Laser powder bed fusion	The experimental group showed enhanced bone formation compared with pure Zn counterparts	Zn-1Mg porous scaffolds presented promising results to fulfill customized requirements of biodegradable bone implants.
Remy M. et al., 2021 [64]	n = 30 male SD rats	-Group with β TCP scaffold (n = 5) -Group with β TCP scaffold and collagen (n = 5)	-Group with β-TCP/collagen/empty vector (n = 5) -Group with β-TCP/pDNA 5 μg miRNA 200c (n = 5) -Group with β-TCP/collagen/pDNA 1 μg miRNA-200c (n = 5) -Group with β-TCP/collagen/pDNA 5 μg miRNA-200c (n = 5)	4 weeks	μ-CT, histology	Stereolithography	-The groups that contained miR-200c demonstrated greater bone formation -Bone formation was higher in the scaffold containing βTCP/collagen/pDNA 5 μg miR-200c	Incorporation of miR increases scaffold efficacy in bone regeneration
Rogowska-Tylman J. et al., 2019 [113]	n = 15 male rabbits	-Group with β TCP scaffold -Group with PCL scaffold	-Group with β-TCP/ HA scaffold -Group with PCL/HA scaffold	3 months	μ-CT, histology, immunohistochemistry	Foaming process/3D printing	The highest bone growth occurred in the group that had the β-TCP/ HA scaffold, followed by the group with the β TCP	The addition of HA particles increases bone regeneration
Ryu J. et al., 2021 [91]	n = 32 mandibular defects in male beagle dogs	-Group without scaffold (n = 8) -Group with Bio-Oss and rhBMP-2 (n = 12)	Group with HA scaffold/ β-TCP/ rhBMP-2 (n = 12)	6, 12 weeks	Histology, imagiology	Stereolithography	There was no significant difference between the Bio-Oss group and the experimental group	Bone formation is not significantly different with HA scaffold/ β- TCP/ rhBMP-2 or with Bio-Oss particles and rhBMP-2
Seo Y. et al., 2022 [114]	n = 40 bone defects in New Zealand White rabbit	Group without scaffold (n = 10)	-Group with β-TCP/ HA scaffold with 0.8 mm pore diameter (n = 10) -Group with β-TCP/ HA scaffold with 1 mm pore diameter (n = 10) -Group with β-TCP/ HA scaffold with 1.2 mm pore diameter (n = 10)	2, 8 weeks	μ-CT, histology	Stereolithography	Among the experimental groups, the 1.0- and 1.2-mm groups exhibited signifcantly larger areas of new bone compared with the 0.8-mm group	β-TCP/ HA block substitutes with different pore diameter promoted faster bone regeneration than that in the natural healing group
Shim J. et al., 2017 [66]	n = 3 male beagle dogs	Group with collagen membrane (n = 1)	-Group with PCL scaffold (n = 1) -Group with PCL scaffold/ β-TCP (n = 1)	8 weeks	μ-CT, histology	Multilayer membrane 3D printing	PCL/ β-TCP scaffold is more effective than PCL and than collagen membrane in terms of bone regeneration	PCL/ β-TCP scaffold appears to be a more effective alternative to collagen membrane in bone regeneration
Shim J. et al., 2017 [115]	n = 8 New Zealand rabbits	Group without scaffold	-Group with 30% porous PCL membrane -Group with 50% porous PCL membrane -Group with 70% porosity PCL membrane	4 weeks	μ-CT, Histometric Analysis	Extrusion-based 3D printing	-The group with the 30% porosity scaffold showed a higher level of bone formation compared to the experimental groups -The control group obtained more bone formation than the scaffold with 50% porosity	-Bone formation was significantly higher in PCL membranes with low porosity -The PCL membrane with 30% porosity is the most favorable for bone regeneration
Shuai C. et al., 2021 [67]	n = 18 New Zealand rabbits	Group without scaffold (n = 6)	-Group with PLLA/PGA/HA scaffold (n = 6) -Group with PLLA/HA scaffold (n = 6)	4, 8 weeks	μ-CT	Laser-assisted 3D printing	The PLLA/PGA/HA scaffold showed greater osteogenesis and vascularization	PLLA/PGA/HA scaffold is promising for bone regeneration
Tcacencu I. et al., 2018 [68]	n = 15 male SD rats	Group with PLLA scaffold (n = 3)	-Group with glass-ceramic scaffold AW (n = 3) -PLLA/AW scaffold Group (n = 6)	12 weeks	Histology	Indirect 3D printing/fused filament fabrication	-No bone formation was observed in the control group -The highest bone formation occurred in the group with the PLLA/AW scaffold	PLLA/AW scaffold is effective in bone regeneration
Tovar N. et al., 2018 [116]	n = 14 New Zealand rabbits	Group without scaffold (n = 4)	Group with β-TCP scaffold (n = 10)	8, 12, 24 weeks	μ-CT, histology	Extrusion-based 3D printing	-The control group showed limited bone growth -In the experimental group, the amount of bone formed was greater at 12 and 24 weeks	The β-TCP scaffolds are biocompatible, resorbable and can regenerate bone
Tsai C. et al., 2019 [69]	n = 12 New Zealand rabbits	Group with titanium scaffold (n = 6)	Group with titanium/Mg- CS and CH scaffold	6 weeks	Histology	Selective laser melting	Less bone regeneration was observed in the control group	Mineralization was higher in the experimental scaffold, which makes it promising for bone defect regeneration
Tulyaganov D. et al., 2022 [117]	n = 16 male Chinchilla rabbits	Group with glass powder (n = 8)	Group with robocast glass scaffold (n = 8)	3, 6 months	Histology	Extrusion-based 3D printing	The scaffolds exhibited a clear osteogenic effect upon implantation and underwent gradual resorption followed by ossification	The scaffold is promising in bone tissue engineering and show promise for potential translation to clinical assessment
Ulbrich L. et al., 2021 [118]	n = 120 male Wistar rats	-Group with empty bone defects -Group with autogenous bone -Group with Bio-Oss scaffold -Group with PBAT scaffold	Group with PBAT/BG scaffold	15, 30, 60 days	μ-CT	Fused deposition modeling	PBAT/ BAGNb presented new bone formation comparable to controls	The combination of PBAT and BAGNb may be an alternative to produce bioactive materials with controllable shapes and properties for bone regeneration treatments
Umeyama R. et al., 2020 [70]	C57BL/6J male rats	Group with β-TCP/RCP scaffold	Group with β-TCP/RCP scaffold and bone marrow cells cultured in an osteogenic environment for 4, 7, and 14 days	8 weeks	Histology	3D printing	The group with the scaffold whose cells had been cultured in an osteogenic environment for 7 days showed the highest osteogenic potential	Bone marrow cells should be cultured in osteogenic medium for 7 days before integrating β-TCP/RCP scaffold
Van hede D. et al., 2021 [119]	n = 16 Wistar male rats	-CaP matrix with orthogonal geometry -CAP matrix + Bio-Oss	Group with CaP matrix with gyroid geometry	4, 8 weeks	μ-CT	Stereolithography	In the group with the gyroid scaffold, greater bone formation was observed at 4 and 8 weeks	Gyroid geometry is promising for bone regeneration
Wang M. et al., 2019 [120]	n = 16 New Zealand rabbits	Group with autologous bone graft (n = 8)	Group with β-TCP scaffold and dipyridamole (n = 8)	24 weeks	Histology	3D printing	The group with the experimental scaffold demonstrated greater bone regeneration	The β-TCP and dipyridamole scaffold is promising in bone defect regeneration
Wang P. et al., 2021 [71]	n = 72 SD female rats	Group with PLLA scaffold (n = 8)	-Group with PLLA scaffold conditioned with sodium hydroxide (n = 8) -Group with PLLA scaffold with PDA conditioned with sodium hydroxide (n = 8)	4, 8 weeks	μ-CT, histology	Fused deposition modeling	Bone formation at weeks 4 and 8 was higher in the group with the scaffold with PDA, followed by the PLLA scaffold conditioned with sodium hydroxide	The presence of PDA increases osteogenesis in the scaffold
Wang S. et al., 2020 [72]	n = 12 female BALB/c mice	Group with PCL/Bio-Oss scaffold (n = 6)	Group with PCL/Bio-Oss/NaOH scaffold (n = 6)	8 weeks	Histology	Fused deposition modeling	In the group with the PCL/Bio-Oss/NaOH scaffold, a greater bone formation was observed	NaOH treatment increased the hydrophilicity of the scaffold by increasing the osteogenic properties
Won J. et al., 2016 [92]	n = 3 male beagle dogs	Group with collagen membrane	Group with PCL/PLGA/β-TCP and Bio-Oss scaffold	8 weeks	μ-CT, histology	Extrusion-based 3D printing	-Bone formation was similar in both groups -The scaffold of the experimental group showed better mechanical properties	The PCL/PLGA/β-TCP scaffold promotes bone regeneration levels similar to collagen membrane, but has better mechanical properties
Wu Y. et al., 2019 [73]	Wistar rats	Group with SC/PCL scaffold	Group with dECM/SC/PCL scaffold	4 weeks	μ-CT	Extrusion-based 3D printing	Bone regeneration was superior in the dECM/SC/PCL group	Decellularization combined with 3D scaffolds can be applied in bone regeneration
Xia D. et al., 2022 [74]	n = 15 New Zealand rabbits	Group with zinc scaffold	Group with pure zinc porous scaffold	4, 12, 24 weeks	μ-CT	Laser powder bed fusion technology	Bone regeneration was superior in the group with pure zinc porous scaffold	Pure Zn porous scaffolds with customized structures represent a promising biodegradable solution for treating large bone defect
Xu Z. et al., 2019 [75]	n = 6 BALB/c mice	Group without scaffold	-Group with PLGA/ β -TCP scaffold -Group with PLGA scaffold/ β -TCP/1 mg polydopamine -Group with PLGA scaffold / β -TCP/2 mg polydopamine	2, 6 weeks	μ-CT, histology	Extrusion-based 3D printing	The higher the PDA concentration, the greater the bone regeneration at 2 and 6 weeks	The addition of PDA allows for good results, and has a lot of potential in bone regeneration
Yu L. et al., 2020 [121]	n = 18 SD rats	Group with Ti scaffold	-Group with Ti and MSC scaffold -Group with Ti scaffold and RA	8 weeks	μ-CT, histology	3D printing	-In the control group, bone formation was almost null -The greatest bone regeneration occurred in the group with RA	The combination of pluripotent stem cells and Ti scaffolds with RA can be used to repair bone defects
Yun J. et al., 2019 [89]	n = 12 beagles	Group without scaffold	-Group with PLLA/PLGA/HA scaffold -Group with PLLA/PLGA/HA/BMP-2 scaffold	20 weeks	μ-CT, histology, imagiology	Extrusion-based 3D printing	-The PLLA/PLGA/HA scaffold is biodegradable and was replaced by bone -Bone regeneration was significantly higher in the group with BMP-2	Bone defects can be successfully treated with PLLA/PLGA/HA/BMP-2
Yun S. et al., 2021 [77]	n = 27 SD rats	Group without scaffold (n = 3)	-Group with dECM scaffold (n = 8) -Group with β TCP scaffold (n = 8) -Group with dECM/ β TCP scaffold (n = 8)	4 weeks	μ-CT, histology	Extrusion-based 3D printing	The group with the dECM/ β TCP scaffold showed greater bone formation	The dECM/ β TCP scaffold has ideal osteogenic potential to treat bone defects
Zhang W. et al., 2017 [122]	n = 38 male New Zealand rabbits	Group with β-TCP scaffold (n = 12)	-Group with BRT scaffold (n = 12) -Group with BRT-H scaffold (n = 14)	4, 12 weeks	μ-CT	Extrusion-based 3D printing	The group with the BRT-H scaffold promoted significantly more bone regeneration	BRT-H scaffold is promising in the repair of large bone defects
Zhang Y. et al., 2019 [79]	n = 24 male Wistar rats	Group without scaffold (n = 6)	-Group with β TCP/ PLGA/ OG /BMP- 2 (n = 6) -Group with β TCP/ PLGA/OG (n = 6) -Group with β TCP/ PLGA (n = 6)	4, 12 weeks	μ-CT, histology	Extrusion-based 3D printing	In the group with β TCP/ PLGA/OG/BMP- 2 the highest bone formation was observed, followed by the group with β TCP/PLGA/OG and β TCP/PLGA	BMP-2 peptide and OG are favorable for bone growth and enhance bone regeneration, making PTG/P scaffold promising in the repair of bone defects
Zhang Z. et al., 2021 [33]	n = 12 New Zealand rabbits	Group with p-Ta scaffold (n = 6)	Group with p-Ta-nt scaffold (n = 6)	2 weeks	histology	3D printing laser melting system	Bone formation was significantly higher in the experimental group	Tantalum matrices with nanotubes show promise in bone regeneration
Zhong L. et al., 2020 [81]	n = 24 male SD rats	Group without scaffold (n = 6)	-Group with PCL scaffold (n = 6) -Group with PCL/DCPD scaffold (n = 6) -Group with PCL/DCPD scaffold/ nanoZIF-8 (n = 6)	12 weeks	μ-CT	Extrusion-based 3D printing	The group with the PCL/DCPD/nanoZIF-8 scaffold induced significantly more bone formation	NanoZIF-8 has great potential in treating bone defects

ADSCs—adipose tissue derived stem cells, Asp@Lipo—aspirin loaded liposomes, AW—apatite/volastonite, BFP-1—bone forming peptide 1, Bio-Oss—deproteinized bovine bone minerals, BM-MSCs—bone marrow derived mesenchymal stem cells, BMP-2—bone morphogenetic protein-2, BRT—β tricalcium phosphate, silicon, magnesium, and calcium, BRT-H—β tricalcium phosphate, silicon, magnesium, and calcium with hollow pipe structure, CaP—calcium phosphate, CGRP—hydroxyapatite/sodium alginate/calcitonin gene-related peptide, CH—chitosan, CS—calcium sulfate, DCPD—calcium phosphate dihydrate, dECM—decellularized extracellularized matrix, dECM—decellularized extracellular matrix, ECM—natural-like extracellular matrix, ETG—sodium hydroxide-conditioned polylactic acid, EV—extracellular vesicle, FP—black phosphorus, Gel/SF—gelatin/silk fibrin, Gel/SF/0Cu-BG—silk gelatin/fibrin and bioactive glass, Gel/SF/10Cu-BG—silk gelatin/fibrin/bioactive glass and 15% copper, Gel/SF/2Cu-BG—silk gelatin/fibrin/bioactive glass and 7% copper, Gel/SF/5Cu-BG—silk gelatin/fibrin/bioactive glass and 10% copper, GTA—glutaraldehyde, HA—hydroxyapatite, HCCS-PDA—calcium silicate and hydroxyapatite collagen with polydopamine binding, hGMSCs—human gum mesenchymal stem cells, hSF-MSCs—synovial mesenchymal stem cells, KR-34893—bioactive organic compound, LayFomm—polyvinyl acid + polyurethane, mg—milligram, Mg- CS—calcium silicate, MgO—magnesium oxide, MgP—magnesium phosphate, miRNA—microRNA, MSC—mesenchymal stem cells, NG—naringin, OG—graphene oxide, PBAT—poly(butylene adipate-co-terephthalate), PCL—polycaprolactone, PCL/T0/B2—polycaprolactone/human recombinant bone protein type 2, PCL/T50—ratio 1:1 polycaprolactone / β tricalcium phosphate, PCL/T50/B2—polycaprolactone/β tricalcium phosphate/human recombinant bone protein type 2, PCLD—dopamine-immersed polycaprolactone, PCLDB100—dopamine-immersed polycaprolactone and BFP-1 at 100 ug/mL, PCLDB1000—dopamine-immersed polycaprolactone and BFP-1 at 1000 ug/mL, pDNA—DNA plasmid, PEEK—polyetherketone, PEG—polyethylene glycol, PEI-EVs—polylactic acid/extracellular vesicle with polyethyleneimine, PGA—polyglycolic acid, PLGA—poly(lactic acid-co-glycolic acid), PPG-1. 5—polyacrylamide, polyurethane, PRF—platelet-rich fibrin, PRP—platelet-rich plasma, p-Ta-nt—tantalum with nanotubes, PTFE—polytetrafluoroethylene, PTG—polylactic acid with polydopamine conditioned with sodium hydroxide, qPCR—real-time polymerase chain reaction, RA—retinoic acid, RCP—recombinant collagen peptide, rhBMP-2—human recombinant bone protein type 2, SA—sodium alginate, SC—calcium silicate, SD—Sprague Dawley, SiO_2_—silica, SrSC—calcium strontium silicate, Ti—titanium, β-TCP—β-tricalcium phosphate, μ-CT—microcomputed tomography.

**Table 3:** Biomaterials described in the included studies (*in vitro* and *in vivo*).

		*In Vitro* Studies	*In Vivo* Studies
**Biomaterials**	β-TCP	16	27
	PCL	16	20
	HA	7	16
	PLLA	7	6
	CS	4	6
	Collagen	4	5
	PLGA	4	5
	dECM	3	5
	Hydrogel	5	3
	MgP	2	2
	Zn-1Mg	0	4
	BG	3	1
	PDA	3	0
	MgO	2	1
	HCCS-PDA	1	2
	Ti	1	1
	PVA	2	0
	OG	1	1
	p-Ta-nt	1	1
	nanoZIF-8	1	1
	DCPD	1	1
	Layform	1	1
	Sodium alginate	1	1
	Gelatin	1	1
	SiO	1	1
	PEEK	1	1
	PGA	1	1
	AW	1	1
	Gel/SF	0	1
	CaP matrix	0	1
	Robocast glass	0	1
	PEI-EVs	0	1
	PTFE	0	1
	Polyvinyl acid	0	1
	PEG	0	1
	PCLD	0	1
	SA	0	1
	Graphene	0	1
	Akermanite	0	1
	Ti_3_C_2_ MXene	1	1
	FP nanoparticles	0	1
	PBAT	0	1
	Polydopamine	0	1
	BRT	0	1
	GCP	1	0
	Bioactive Silk Fibrin Glass	1	0
**Biomolecules**	RhBMP-2	6	7
	Dipyridamole	1	4
	PRF	0	3
	hSF-MSCs	1	2
	miRNA	2	1
	NaOH	2	1
	Curcumin	0	1
	RGD	0	1
	Asp@Lipo	1	1
	BFP-1	1	1
	RCP	1	1
	VEGF	1	1
	Heparin	0	1
	ADSCs	0	1
	NG	0	1
	CGRP	0	1
	BM-MSCs	0	1
	pDNA	0	1
	DPSCs	1	0
	Dexamethasona	1	0
	Glycerol	1	0
	KR-34893	1	0
	PRP	1	0

**Table 4:** Analysis of evaluation methods in *in vitro* and *in vivo* studies.

Study Type	µ-CT	Histology	qRT-PCR (Osteogenesis-Related Gene Expression)	p-Nitrophenol Assay (ALP Activity)	AKT Assay Kit (ALP Activity)	Imagiology	Western-Blot (Expression Levels of Osteogenic-Related Proteins)	Imunohistochemistry	Western-Blot (ALP Activity)	ELISA (Osteogenic-Related Protein Secretion)
*In vitro*	0	1	27	9	7	0	2	0	1	1
*In vivo*	57	56	3	0	0	4	0	2	0	0

**Table 5:** Analysis of biomaterials 3D printing techniques in *in vitro* and *in vivo* studies.

Study Type	Extrusion Based Bioprinting	Fused Deposition Modeling	3D Printing (No Specific Method)	Stereolithograhy	Laser Sintering Technique	Digital Light Processing Type 3D Printing System	Selective Laser melting	Laser Powder Bed Fusion	Inkjet-Based Bioprinting	Microvalve Bioprinting	Extrusion-Based Hydrogel	Nanosheets via Liquid Phase Stripping Method	Multilayer Membrane 3D Printing	Indirect 3D Printing/Fused Filament Fabrication	Binder Jetting	Direct Ink Writing Technique	Micro Extrusion	Foaming Process/3D Printing	Electrospinning and Electrospraying	3D Printed Scaffolds
*In vitro*	23	6	6	2	3	1	2	1	2	1	1	1	1	1	0	0	0	0	0	0
*In vivo*	27	10	8	7	3	3	2	2	1	1	1	1	1	1	1	1	1	1	1	1

**Table 6:** Risk of bias of *in vitro* studies.

	Structured Summary	Scientific Background and Explanation of Rationale	Specific Objectives and/or Hypotheses	Intervention for Each Group	Outcome	Sample Size	Random Allocation	Allocation Concealment Mechanism	Implementation	Blinding	Statistical Methods	Outcomes and Estimation	Limitations	Funding	Protocol
Alksne M. et al., 2020 [34]	Y	Y	Y	Y	Y	Y	N	N	N	N	Y	Y	Y	Y	Y
Bae E. et al., 2018 [35]	Y	Y	Y	Y	Y	N	Y	Y	N	Y	Y	Y	Y	Y	N
Cao Y. et al., 2019 [36]	Y	Y	Y	Y	Y	N	N	N	N	N	Y	Y	N	Y	N
Chen Y. et al., 2018 [37]	Y	Y	Y	Y	Y	N	N	N	N	N	Y	Y	N	Y	N
Chiu Y. et al., 2019 [38]	Y	Y	Y	Y	Y	N	N	N	N	N	Y	Y	N	Y	N
Cooke M. et al., 2020 [39]	Y	Y	Y	Y	Y	N	N	N	N	N	Y	Y	Y	Y	N
Dai Q. et al., 2021 [40]	Y	Y	Y	Y	Y	N	N	N	N	N	Y	Y	N	Y	N
Dubey N. et al., 2020 [41]	Y	Y	Y	Y	Y	N	Y	N	N	N	Y	Y	Y	N	N
Fahimipour F. et al., 2019 [42]	Y	Y	Y	Y	Y	N	N	N	N	N	Y	Y	Y	Y	N
Gómez-Cerezo M. et al., 2020 [43]	Y	Y	Y	Y	Y	N	N	N	N	N	Y	Y	N	Y	N
Han L. et al., 2021 [44]	Y	Y	Y	Y	Y	N	Y	Y	N	N	Y	Y	Y	Y	N
Huang K. et al., 2021 [45]	Y	Y	Y	Y	Y	N	N	N	N	N	Y	Y	Y	Y	Y
Jeong J. et al., 2020 [46]	Y	Y	Y	Y	Y	N	N	N	N	N	Y	Y	N	Y	N
Kao C. et al., 2015 [47]	Y	Y	Y	Y	Y	N	N	N	N	N	Y	Y	N	Y	N
Ke, D. et al., 2018 [48]	Y	Y	Y	Y	Y	N	N	N	N	N	Y	Y	N	Y	N
Kim B. et al., 2018 [49]	Y	Y	Y	Y	Y	N	N	N	N	Y	Y	Y	N	Y	N
Kim J. et al., 2017 [50]	Y	Y	Y	Y	Y	N	N	N	N	N	Y	Y	N	Y	N
Lee S. et al., 2018 [51]	Y	Y	Y	Y	Y	Y	Y	Y	N	N	Y	Y	N	Y	N
Li J. et al., 2017 [52]	Y	Y	Y	Y	Y	N	Y	N	N	N	Y	Y	Y	Y	Y
Li Y. et al., 2019 [53]	Y	Y	Y	Y	Y	N	N	N	N	N	Y	Y	N	Y	Y
Lin Y. et al., 2019 [54]	Y	Y	Y	Y	Y	N	N	N	N	N	Y	Y	N	N	Y
Lin YH. et al., 2017 [55]	Y	Y	Y	Y	Y	N	N	N	N	N	Y	Y	N	S	N
Lin YH. et al., 2019 [56]	Y	Y	Y	Y	Y	N	N	N	N	N	Y	Y	N	Y	Y
Martin V. et al., 2019 [57]	Y	Y	N	Y	Y	N	N	N	N	N	Y	Y	N	Y	N
Mi X. et al., 2022 [58]	Y	Y	Y	Y	Y	N	Y	N	N	N	Y	Y	Y	Y	N
Miao Y. et al., 2019 [59]	Y	Y	Y	Y	Y	N	N	N	N	N	Y	Y	N	Y	N
Midha S. et al., 2018 [60]	Y	Y	Y	Y	Y	N	N	N	N	N	Y	Y	Y	Y	N
Pan T. et al., 2022 [61]	Y	Y	Y	Y	Y	N	Y	N	N	N	Y	Y	Y	Y	N
Park J. et al., 2015 [32]	Y	Y	Y	Y	Y	N	Y	N	N	N	Y	Y	N	Y	N
Park S. et al., 2020 [62]	Y	Y	Y	Y	Y	N	N	N	N	N	Y	Y	N	Y	Y
Ratheesh. G. et al., 2021 [63]	Y	Y	Y	Y	Y	N	N	N	N	N	Y	Y	Y	Y	N
Remy M. et al., 2021 [64]	Y	Y	Y	Y	Y	N	N	N	N	N	Y	Y	Y	S	N
Roh H. et al., 2016 [65]	Y	Y	Y	Y	Y	N	Y	N	N	N	Y	Y	N	Y	N
Shim J. et al 2017 [115]	Y	Y	Y	Y	Y	N	Y	N	N	N	Y	N	N	Y	N
Shuai C. et al., 2020 [67]	Y	Y	Y	Y	Y	N	N	N	N	N	Y	Y	N	Y	N
Tcacencu I. et al., 2018 [68]	Y	Y	Y	Y	Y	N	Y	N	N	N	Y	Y	N	Y	N
Tsai C. et al., 2019 [69]	Y	Y	Y	Y	Y	N	N	N	N	N	Y	Y	N	Y	N
Umeyama R. et al., 2020 [70]	Y	Y	Y	Y	Y	N	N	N	N	N	Y	Y	N	Y	N
Wang P. et al., 2021 [71]	Y	Y	Y	Y	Y	N	Y	N	N	N	Y	Y	N	Y	N
Wang S. et al., 2020 [72]	Y	Y	Y	Y	Y	N	N	N	N	N	Y	Y	Y	Y	N
Weinand C. et al., 2006 [31]	Y	Y	Y	Y	Y	N	N	N	N	N	Y	N	Y	N	N
Wu Y. et al., 2019 [73]	Y	Y	Y	Y	Y	N	N	N	N	N	Y	N	N	Y	N
Xia D. et al., 2022 [74]	Y	Y	Y	Y	Y	N	N	N	N	N	Y	Y	Y	Y	Y
Xu Z. et al., 2019 [75]	Y	Y	Y	Y	Y	N	N	N	N	N	Y	N	N	Y	N
Xu Z. et al., 2022 [76]	Y	Y	Y	Y	Y	N	Y	N	N	N	Y	Y	N	Y	N
Yun S. et al., 2021 [77]	Y	Y	Y	Y	Y	N	Y	N	N	N	Y	N	Y	Y	N
Zamani Y. et al., 2021 [78]	Y	Y	Y	Y	Y	N	Y	N	N	N	Y	Y	N	Y	N
Zhang Y. et al., 2019 [79]	Y	Y	Y	Y	Y	N	Y	N	N	N	Y	Y	N	Y	N
Zhang Z. et al., 2021 [33]	Y	Y	Y	Y	Y	N	Y	N	N	N	Y	Y	Y	Y	N
Zhong L. et al., 2020 [81]	Y	Y	Y	Y	Y	N	Y	N	N	N	Y	Y	Y	Y	N
Zhao N. et al., 2017 [80]	Y	Y	Y	Y	Y	N	N	N	N	N	Y	N	Y	Y	N

Y—Yes; N—No.

**Table 7:** Risk of bias of *in vivo* studies.

	Sequence Generation	Baseline Characteristics	Allocation Concealment	Random Housing	Blinding	Random Outcome Assessment	Blinding	Incomplete Outcome Data	Selective Outcome Reporting	Other Sources of Bias
Bae E. et al., 2018 [35]	N	Y	N	Y	N	Y	Y	Y	Y	Y
Bekisz J. et al., 2018 [93]	N	Y	N	N	N	Y	N	Y	Y	Y
Bose S. et al., 2018 [85]	N	Y	N	U	N	Y	N	Y	Y	Y
Chang P. et al., 2021 [94]	N	Y	N	N	N	Y	N	Y	Y	Y
Chen M. et al., 2021 [95]	N	Y	N	N	N	Y	N	Y	Y	Y
Chiu Y. et al., 2019 [38]	N	Y	N	N	N	Y	N	Y	Y	Y
Cooke M. et al., 2020 [39]	N	Y	N	U	N	Y	N	Y	Y	Y
Dai Q. et al., 2021 [40]	N	Y	N	N	N	Y	N	Y	Y	Y
Diomede F. et al., 2018 [96]	N	Y	N	Y	N	Y	N	Y	Y	Y
Dubey N. et al., 2020 [41]	N	Y	N	N	N	Y	N	Y	Y	Y
El-Habashy S. et al., 2021 [97]	N	Y	N	N	N	Y	N	Y	Y	Y
Fahimipour F. et al., 2019 [42]	N	Y	N	Y	N	Y	N	Y	Y	Y
Fama C. et al., 2020 [98]	U	N	N	N	N	N	N	N	N	Y
Guéhennec L. et al., 2019 [90]	N	Y	N	N	N	Y	N	Y	Y	N
Han L. et al., 2021 [44]	N	Y	N	N	N	Y	N	Y	Y	Y
He M. et al., 2021 [99]	N	Y	N	N	N	Y	N	Y	Y	Y
Huang K. et al., 2021 [45]	N	Y	N	N	N	Y	N	Y	Y	Y
Ishack S. et al., 2017 [88]	N	Y	N	N	N	Y	N	Y	Y	Y
Jeong J. et al., 2020 [46]	N	Y	N	N	N	Y	N	Y	Y	Y
Jia L. et al., 2021 [100]	N	Y	N	Y	N	Y	N	Y	Y	Y
Johnson Z. et al., 2021 [101]	N	Y	N	N	N	Y	N	Y	Y	Y
Ke D. et al., 2018 [48]	N	Y	N	Y	N	Y	N	Y	Y	Y
Kim J. et al., 2020 [102]	N	Y	N	N	N	Y	N	Y	Y	Y
Kim J. et al., 2017 [50]	N	Y	N	N	N	Y	N	Y	Y	Y
Lee D. et al., 2018 [103]	N	Y	N	N	N	Y	N	Y	Y	Y
Lee J. et al., 2021 [86]	N	Y	N	N	N	Y	N	Y	Y	Y
Lee S. et al., 2019 [51]	N	Y	N	N	N	Y	N	Y	Y	Y
Lee SH. et al., 2019 [87]	N	Y	N	N	N	Y	N	Y	Y	Y
Liang T. et al., 2021 [104]	N	Y	N	Y	N	Y	N	Y	Y	Y
Li J. et al., 2017 [52]	N	Y	N	N	N	Y	N	Y	Y	Y
Li Y. et al., 2019 [53]	N	Y	N	N	N	Y	N	Y	Y	Y
Lim H. et al., 2020 [105]	N	Y	N	N	N	Y	N	Y	Y	Y
Lin YH. et al., 2019 [56]	N	Y	N	N	N	Y	N	Y	Y	Y
Lin YH. et al., 2017 [55]	N	Y	N	N	N	Y	N	Y	Y	Y
Liu A. et al., 2016 [106]	N	Y	N	N	N	Y	N	Y	Y	Y
Lopez C. et al., 2019 [107]	N	Y	N	N	N	Y	Y	Y	Y	Y
Mi X. et al., 2022 [58]	N	Y	N	N	N	Y	N	Y	Y	Y
Miao Y. et al., 2019 [59]	N	N	N	N	N	Y	N	Y	Y	Y
Naudot M. et al., 2020 [108]	N	Y	N	U	N	Y	N	Y	Y	Y
Pan T. et al., 2022 [61]	N	Y	N	N	N	Y	N	Y	Y	Y
Park S. et al., 2020 [62]	N	Y	N	U	N	Y	N	Y	Y	Y
Park J. et al., 2015 [32]	N	Y	N	N	N	Y	N	Y	Y	Y
Pae H. et al., 2018 [109]	N	Y	N	Y	N	Y	N	Y	Y	Y
Qiao S. et al., 2020 [110]	N	Y	N	N	N	Y	N	Y	Y	Y
Qin H. et al., 2022 [111]	N	Y	N	Y	N	Y	N	Y	Y	N
Qin Y. et al., 2022 [112]	N	Y	N	Y	N	Y	N	Y	Y	Y
Remy M. et al., 2021 [64]	N	Y	N	N	N	Y	N	Y	Y	Y
Rogowska-Tylman J. et al., 2019 [113]	N	Y	N	N	N	N	N	N	Y	Y
Ryu J. et al., 2021 [91]	N	Y	N	N	N	Y	Y	Y	Y	N
Seo Y. et al., 2022 [114]	N	Y	N	Y	N	Y	N	Y	Y	Y
Shim J. et al., 2017 [115]	N	Y	N	N	N	Y	N	Y	Y	Y
Shim J. et al., 2018 [66]	N	Y	N	Y	N	Y	Y	Y	Y	N
Shuai C. et al., 2021 [67]	N	Y	N	N	N	Y	N	Y	Y	Y
Tcacencu I. et al., 2018 [68]	N	Y	N	N	N	Y	N	Y	Y	Y
Tovar N. et al., 2018 [116]	N	Y	N	N	N	Y	N	Y	Y	N
Tsai C. et al., 2019 [69]	N	N	N	N	N	Y	N	Y	Y	Y
Tulyaganov D. et al., 2022 [117]	N	Y	N	Y	N	Y	N	Y	Y	Y
Ulbrich L. et al., 2021 [118]	Y	Y	N	Y	N	Y	N	Y	Y	Y
Umeyama R. et al., 2020 [70]	N	U	N	N	N	Y	N	Y	Y	N
Van hede D. et al., 2021 [119]	N	N	N	N	N	Y	N	Y	Y	N
Wang M. et al., 2019 [120]	N	Y	N	N	N	Y	Y	Y	Y	Y
Wang P. et al., 2021 [71]	N	Y	N	N	N	Y	N	Y	Y	Y
Wang S. et al., 2020 [72]	N	Y	N	U	N	Y	N	Y	Y	Y
Won J. et al., 2016 [92]	N	Y	N	N	N	Y	Y	Y	Y	Y
Wu Y. et al., 2019 [73]	N	N	N	Y	N	Y	N	Y	Y	Y
Xia D. et al., 2022 [74]	N	Y	N	Y	N	Y	N	Y	Y	Y
Xu Z. et al., 2019 [76]	N	N	N	N	N	Y	N	Y	Y	Y
Yu L. et al., 2020 [121]	N	N	N	N	N	Y	N	Y	Y	Y
Yun J. et al., 2019 [89]	N	Y	N	N	N	Y	N	Y	Y	Y
Yun S. et al., 2021 [77]	N	N	N	Y	N	Y	N	Y	Y	Y
Zhang W. et al., 2017 [122]	N	Y	N	N	N	Y	N	Y	Y	Y
Zhang Y. et al., 2019 [79]	N	Y	N	N	N	Y	N	Y	Y	Y
Zhang Z. et al., 2021 [33]	N	N	N	N	N	Y	N	Y	Y	Y
Zhong L. et al., 2020 [81]	N	Y	N	N	N	Y	N	Y	Y	Y

Y—Yes; N—No; U—unclear.

## Data Availability

The data presented in this study are available on request from the corresponding author.

## Appendix A

**Database**	**Search Phrase**
*Pubmed via Medline and Cochrane Library*	(“Printing, Three-Dimensional” [Mesh] OR “Printing, Three Dimensional” OR “Printings, Three-Dimensional” OR “Three-Dimensional Printings” OR “3-Dimensional Printing*” OR “3 Dimensional Printing*” OR “Printing, 3-Dimensional” OR “Printings, 3-Dimensional” OR “3-D Printing*” OR “3 D Printing*” OR “Printing, 3-D” OR “Printings, 3-D” OR “Three-Dimensional Printing” OR “Three Dimensional Printing” OR “3D Printing*” OR “Printing, 3D” OR “Printings, 3D”) AND (“Bone Regeneration”[Mesh] OR “Bone Regenerations*” OR “Regeneration, Bone” OR “Regenerations, Bone” OR Osteoconduction OR “Alveolar Bone Grafting”[Mesh] OR “alveolar bone grafting*” OR “Alveolar Cleft Grafting” OR “bone graft*” OR “Bone Substitutes”[Mesh] OR “bone substitute*” OR “Replacement Material, Bone” OR “Replacement Materials, Bone” OR “Materials, Bone Replacement” OR “Substitute, Bone” OR “Substitutes, Bone” OR “Bone Replacement Material*” OR “Material, Bone Replacement” ) AND (Dentistry[Mesh] OR dentistry OR oral* OR orofacial OR dental* OR maxillofacial OR “Surgery, Oral”[Mesh] OR “surgery, oral” OR “Maxillofacial Surgery” OR “Surgery, Maxillofacial” OR “Oral Surgery” OR “Cleft Palate”[Mesh] OR “cleft palate*” OR “Palate, Cleft” OR “Palates, Cleft” OR “Cleft Palate, Isolated”)
*Web of Science Core Collection (WOS)*	*TS = (“Print**, *Three Dimensional” OR “Three-Dimensional Print*” OR “3-Dimensional Print*” OR “3 Dimensional Print*” OR “Print**, *3-Dimensional” OR “3-D Print*” OR “3D Print*” OR “Print**, *3-D” OR “ Print**, *3D”) AND TS = ( “Regenerati**, *Bone” OR “Bone Regenerati*” OR osteoconduction OR “Alveolar Bone Graft*” OR “alveolar cleft grafting“ OR “bone graft*” OR “Replacement Material**, *Bone” OR “Material**, *Bone Replacement” OR “Substitute**, *Bone” OR “Bone Replacement Material*” OR “ Material*, *Bone Replacement” OR “bone substitute*”) AND TS = (dent* OR oral* OR orofacial OR maxillofacial OR “Surgery*, *Oral” OR “oral surgery”)*
*EMBASE*	(‘*printing*, *three dimensional’/exp OR ‘printing*, *three dimensional’ OR ‘printings*, *three-dimensional’ OR ‘three-dimensional printings’ OR ‘3-dimensional printing*’ OR ‘3 dimensional printing*’ OR ‘printing*, *3-dimensional’ OR ‘printings*, *3-dimensional’ OR ‘3-d printing*’ OR ‘3 d printing*’ OR ‘printing*, *3-d’ OR ‘printings*, *3-d’ OR ‘three-dimensional printing’/exp OR ‘three-dimensional printing’ OR ‘three dimensional printing’/exp OR ‘three dimensional printing’ OR ‘3d printing*’ OR ‘printing*, *3d’ OR ‘printings*, *3d’) AND (‘bone regeneration’/exp OR ‘bone regeneration’ OR ‘regeneration*, *bone’/exp OR ‘regeneration*, *bone’ OR ‘regenerations*, *bone’ OR ’osteoconduction’/exp OR osteoconduction OR ‘alveolar bone grafting’/exp OR ‘alveolar bone grafting’ OR ‘alveolar cleft grafting’ OR ‘bone graft*’ OR ‘bone graft’/exp OR ‘bone graft’ OR ‘bone transplantation’/exp OR ‘bone transplantation’ OR ‘bone prosthesis’/exp OR ‘bone prosthesis’ OR ‘bone substitute*’ OR ‘replacement material*, *bone’ OR ‘replacement materials*, *bone’ OR ‘materials*, *bone replacement’ OR ‘substitute*, *bone’ OR ‘substitutes*, *bone’ OR ‘bone replacement material*’ OR ‘material*, *bone replacement’) AND (dentistry OR ‘dentistry’/exp OR ‘dentistry’ OR oral OR orofacial OR ‘dental’/exp OR dental OR maxillofacial OR ‘oral surgery’/exp OR ‘oral surgery’)*
